# Computer Simulation of TSP1 Inhibition of VEGF–Akt–eNOS: An Angiogenesis Triple Threat

**DOI:** 10.3389/fphys.2018.00644

**Published:** 2018-05-30

**Authors:** Hojjat Bazzazi, Yu Zhang, Mohammad Jafarnejad, Jeffrey S. Isenberg, Brian H. Annex, Aleksander S. Popel

**Affiliations:** ^1^Department of Biomedical Engineering, School of Medicine, Johns Hopkins University, Baltimore, MD, United States; ^2^Heart, Lung, Blood and Vascular Medicine Institute, University of Pittsburgh, Pittsburgh, PA, United States; ^3^Division of Pulmonary, Allergy, and Critical Care Medicine, Department of Medicine, University of Pittsburgh, Pittsburgh, PA, United States; ^4^Division of Cardiovascular Medicine, Department of Medicine, Robert M. Berne Cardiovascular Research Center, University of Virginia School of Medicine, Charlottesville, VA, United States

**Keywords:** TSP1, CD47, VEGFR2, systems biology, systems pharmacology, computational modeling

## Abstract

The matricellular protein thrombospondin-1 (TSP1) is a potent inhibitor of angiogenesis. Specifically, TSP1 has been experimentally shown to inhibit signaling downstream of vascular endothelial growth factor (VEGF). The molecular mechanism of this inhibition is not entirely clear. We developed a detailed computational model of VEGF signaling to Akt–endothelial nitric oxide synthase (eNOS) to investigate the quantitative molecular mechanism of TSP1 inhibition. The model demonstrated that TSP1 acceleration of VEGFR2 degradation is sufficient to explain the inhibition of VEGFR2 and eNOS phosphorylation. However, Akt inhibition requires TSP1-induced phosphatase recruitment to VEGFR2. The model was then utilized to test various strategies for the rescue of VEGF signaling to Akt and eNOS. Inhibiting TSP1 was predicted to be not as effective as CD47 depletion in rescuing signaling to Akt. The model further predicts that combination strategy involving depletion of CD47 and inhibition of TSP1 binding to CD47 is necessary for effective recovery of signaling to eNOS. In all, computational modeling offers insight to molecular mechanisms involving TSP1 interaction with VEGF signaling and provides strategies for rescuing angiogenesis by targeting TSP1–CD47 axis.

## Introduction

Vascular endothelial growth factor (VEGF) is a critical regulator of angiogenesis in physiological and pathophysiological states. The VEGF family consists of number of secreted proteins: VEGF-A, VEGF-B, VEGF-C, VEGF-D, VEGF-E, and placental growth factor (PlGF), with VEGF-A being the most widely studied of the group. VEGF plays a crucial role in vasculogenesis and developmental angiogenesis (Shalaby et al., 1995; Carmeliet et al., 1996) and adult vascular permeability and homeostasis (Ku et al., 1993; Lee et al., 2007; Curwen et al., 2008). Dysregulation in VEGF signaling contributes to a wide array of diseases including cancer (Kieran et al., 2012; Claesson-Welsh and Welsh, 2013), wound healing (Bao et al., 2009), age-related macular degeneration (Ferrara, 2010), and peripheral arterial disease (PAD) (Mac Gabhann et al., 2010; Annex, 2013; Boucher and Bautch, 2014; Clegg et al., 2017; Clegg and Mac Gabhann, 2018). The response to VEGF is mediated by its binding to multiple receptors and co-receptors on endothelial cells such as VEGF receptor 2 (VEGFR2) and neuropilin-1 (NRP1). VEGF binding to receptor tyrosine kinase VEGFR2 leads to the activation of downstream signaling pathways including ERK1/2 and PI3K/Akt that induce cellular proliferation, survival, motility, and enhanced vascular permeability (Olsson et al., 2006; Dellinger and Brekken, 2011; Simons et al., 2016), the dominant pathway in post-natal angiogenesis. VEGF-VEGFR2 activation also induces nitric oxide (NO) release as a result of the activation of endothelial nitric oxide synthase (eNOS), substantially contributing to the angiogenic response (Papapetropoulos et al., 1997; Fukumura et al., 2001).

Physiological VEGF signaling is tightly regulated by a balance of promoters and inhibitors of angiogenesis (Folkman, 2004). Among the first identified endogenous inhibitors of angiogenesis was the matricellular protein thrombospondin-1 (TSP1) (Bagavandoss and Wilks, 1990; Good et al., 1990; Taraboletti et al., 1990). TSP1 potently inhibits VEGF signaling at multiple levels. At nanomolar concentrations, TSP1 can directly bind and sequester VEGF (Gupta et al., 1999) or lead to the internalization of TSP1–VEGF complex via binding to the TSP1 receptor LDL-related receptor protein 1 (LRP1) (Greenaway et al., 2007). At these concentrations, TSP1 may also inhibit Akt/eNOS/NO signaling by binding to the cell surface receptor CD36 (Isenberg et al., 2007b). Binding of TSP1 to CD36, a fatty acid translocase, also inhibits its ability to uptake myristate into endothelial cells inhibiting activation of Src kinases and cGMP signaling (Isenberg et al., 2007b). At picomolar concentrations, TSP1 potently inhibits angiogenesis by binding to CD47, an integrin associate glycoprotein membrane receptor (Kaur et al., 2010). CD47 is the necessary TSP1 receptor for the inhibition of signals downstream of NO namely soluble guanylate cyclase (sGC) and cGMP-dependent protein kinase (Isenberg et al., 2006, 2008c). TSP1–CD47 interaction also inhibits eNOS activation and eNOS-dependent endothelial cell vasorelaxation (Bauer et al., 2010). Adding to the empirical evidence is the result that mice deficient in CD47 or TSP1 show enhanced angiogenesis in models of wound healing (Isenberg et al., 2008b).

TSP1–CD47 interaction has been demonstrated to potently inhibit VEGFR2 phosphorylation and Akt activation (Kaur et al., 2010). Indeed, suppression of CD47 or downregulation of its expression rescued VEGFR2 phosphorylation indicating that the anti-angiogenic phenotype initiated by TSP1–CD47 interaction goes beyond mere inhibition of NO signaling pointing toward a role in a more global inhibitory effects (Kaur et al., 2010).

Considering the complexity of receptor-level interactions and downstream signaling, computational models should prove expedient in elucidating and clarifying molecular mechanisms involved in TSP1–CD47 interaction with other receptors and suggest new avenues for experimental investigation. Moreover, computational models may inform and discriminate between various therapeutic modalities targeting TSP1–CD47 signaling.

Recently, computational modeling has been applied to investigate the mechanism of inhibition of VEGF signaling by TSP1–CD47 interaction and also test several therapeutic interventions (Bazzazi et al., 2017). A detailed computational model of TSP1 was also developed within the context of cancer to investigate intervention mimicking the anti-angiogenic activity of TSP1 (Rohrs et al., 2016). In order to be relevant, the computational models require detailed signaling modules that include VEGF-VEGFR2 signaling to downstream pathways such as ERK1/2 and PI3K/Akt, which are subsequently integrated with TSP1–CD47 interaction. Detailed computational models of VEGF-VEGFR2 signaling incorporating detailed receptor dynamics have been developed in the past including signaling to ERK1/2 and calcium (Ca) (Tan et al., 2013b; Zhang et al., 2014; Clegg and Mac Gabhann, 2015; Bazzazi and Popel, 2017), and PI3K/Akt (Tan et al., 2013a).

Extending this to look at a different arm of VEGF signaling pathway and angiogenesis, our aim in this study is to build upon previous models of VEGFR2 signaling and present a fundamentally novel computational model of VEGF signaling to PI3K/Akt that takes into account recent experimental data in endothelial cells demonstrating the critical role of the receptor Axl-1 and Src in transducing VEGFR2 activation signal to PI3K/Akt (Ruan and Kazlauskas, 2012a). We also include a Ca–calmodulin (Ca/CaM) activation module to investigate the activation of eNOS by Ca/CaM and Akt phosphorylation. The inclusion of eNOS activation into our model downstream of VEGFR2 is motivated by experimental data demonstrating that eNOS-deficient mice have compromised angiogenesis in response to VEGF (Fukumura et al., 2001). The model is then utilized to investigate potential molecular mechanisms for the experimentally observed inhibition of signaling to Akt by TSP1 (Kaur et al., 2010) and whether these mechanisms may also predict the inhibition of VEGF-induced eNOS activation (Feliers et al., 2005; Chen et al., 2006). Experimentally, there is scant report in the literature suggesting a role for enhanced degradation of VEGFR2 by TSP1 that may contribute to the inhibitory action of TSP1 on VEGF signaling (Kaur et al., 2011). A previous model confirmed that this is sufficient to explain the inhibition of VEGF signaling to ERK1/2 and Ca (Bazzazi et al., 2017). Here, we utilize a more complex receptor cycling model to evaluate whether enhanced degradation hypothesis is sufficient to explain signal inhibition to both Akt and eNOS or whether other mechanisms should be considered.

Clinically, TSP1–CD47 signaling axis might offer an attractive target for pro-angiogenic interventions in diseases such as PAD (Smadja et al., 2011) and impaired wound healing (Rogers et al., 2014; Soto-Pantoja et al., 2014). Our computational model provides a platform to test whether targeting TSP1 or CD47 might be sufficient in rescuing VEGF signaling to Akt and eNOS; hence, paving the way for further validation of these targets for pro-angiogenic therapy.

## Materials and Methods

The rule-based programming language BioNetGen was used to generate reaction network based on the input biological rules (Hlavacek et al., 2006; Faeder et al., 2009). The final model consisted of 824 species (set of 824 ordinary differential equations) interacting via 5467 elementary biochemical reactions. The text of the BioNetGen file is provided as a supplement with the rules and initial seed species defined. The description of the species, rules, and coarse-grained model of signaling is also provided as a Supplementary File. It should be noted that BioNetGen is used for accurately capturing all the different receptor complexes in the model and for conveniently capturing all the reaction rules. Using BioNetGen also avoids manual formulation of the model that would require *a priori* knowledge of all the intermediate reaction steps. The model is simulated within MATLAB 2015b (MathWorks, Natick, MA, United States) using the Sundials solver suite (Hindmarsh et al., 2005). Optimization to determine parameter values was performed using the direct search algorithm, *patternsearch* as part of MATLAB global optimization toolbox. The set of rules for receptor interaction levels are contained in the text file and the model is also presented in Systems Biology Markup Language (SBML) format. Western blot data from the literature were quantified using ImageJ (Schneider et al., 2012).

## Results

### Model Construction: Receptor-Level Interactions and Downstream Signaling

**FIGURE 1:**
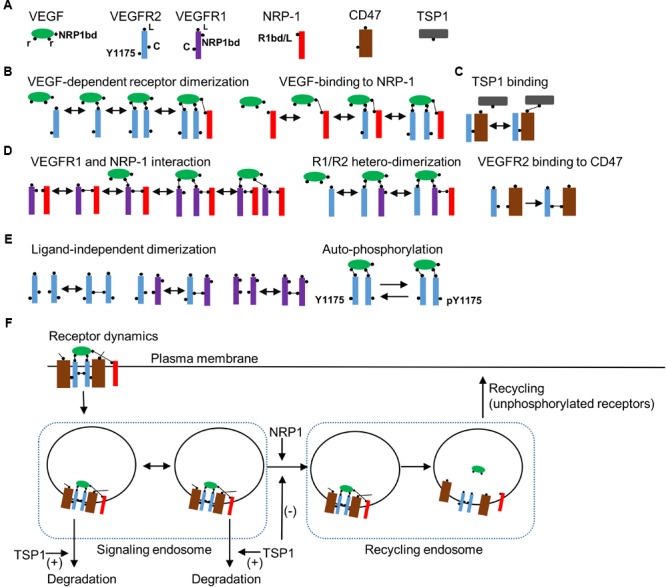
Receptor level rules, species, and dynamics. **(A)** Seed species in the model include the ligand VEGF, VEGFR2, VEGFR1, NRP1, CD47, and TSP1 with binding domains and modification sites indicated in the figure. **(B)** Rules for VEGF-dependent receptor dimerization, VEGF binding to NRP1, and TSP1 binding to CD47 are illustrated. **(C)** Rules for VEGFR1 binding with NRP1, VEGF-mediated VEGFR1 (R1)/VEGFR2 (R2) heterodimerization are shown. **(D)** The rule for the constitutive binding of VEGFR2 to CD47. **(E)** Ligand-independent receptor coupling and receptor autophosphorylation rules. **(F)** Receptor internalization when VEGFR2 is homodimerized by VEGF. The model includes two compartments as signaling endosomes. Transport from signaling endosomes to recycling endosomes occurs when NRP1 is part of the receptor complex. TSP1 may modify the transport of vesicles to the recycling endosome or enhance the degradation of VEGFR2 from the early endosomes. Ligands are dissociated from receptors in recycling endosomes. Only non-phosphorylated receptors recycle back to the membrane from the recycling endosomes.

Detailed receptor interaction module was constructed using a rule-based methodology utilizing BioNetGen similar to previous studies (Hlavacek et al., 2006; Rohrs et al., 2016; Bazzazi et al., 2017; Bazzazi and Popel, 2017). As shown in **Figure 1A**, the seed species include the ligand VEGF-A with two binding sites (r) for the receptor and a single binding site for the coreceptor NRP1 (NRP1bd). VEGFR2 has a ligand-binding site (L), a tyrosine phosphorylation site (represented as Y1175), and a ligand independent coupling site (c).

**FIGURE 2:**
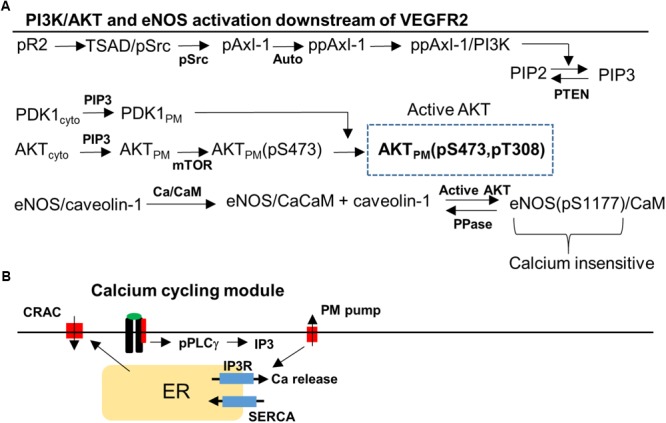
Signaling pathway to Akt, eNOS, and Ca/CaM downstream of VEGFR2. **(A)** Phosphorylated VEGFR2 (pR2) activates pSrc that transduces the signal to PI3K/AKT via Axl-1. Activation of pR2 leads to PLCγ activation that eventually results in Ca elevation and Ca/CaM activation. Ca/CaM is necessary for eNOS dissociation from caveolin-1 and initial activation. Activated Akt phosphorylated eNOS and fully activates it. **(B)** Ca cycling modules that include plasma membrane and SERCA Ca pumps, IP3-sensitive Ca release channels, and CRAC channels.

In effect, we lump together different phosphorylation sites on VEGFR2 to simplify the model. Signaling from distinct phosphorylation site may be incorporated into the BioNetGen description as more experimental data become available. VEGFR1 contains a single binding site for the ligand (L) and another for NRP1 (NRP1bd), and a ligand-independent coupling domain (c). NRP1 has a single-binding site for both the ligand and VEGFR1 (R1bd/L) (Fuh et al., 2000). The main receptor for TSP1 is assumed to be CD47 that has a binding site for VEGFR2 and another for TSP1. The rules for VEGF-dependent receptor dimerization are summarized in **Figure 1B**. TSP1 binds to VEGFR2-coupled CD47 (**Figure 1C**). Biologically, TSP1 is a tetramer with potentially three different binding sites for CD47, but to simplify the model, we assume that TSP1 binding to CD47 is effectively a one-to-one interaction. VEGFR1 and NRP1 interact constitutively as shown in **Figure 1D**. VEGFR1/VEGFR2 heterodimerization is also included along with constitutive binding of VEGFR2 to CD47 (**Figure 1D**). For completeness, model here includes ligand-independent dimerization as shown in **Figure 1E** (Neagoe et al., 2005; Mac Gabhann and Popel, 2007). Engagement of VEGF with two VEGFR2 receptors leads to autophosphorylation of the receptors (**Figure 1E**). Furthermore, VEGFR2 receptors internalize after the homodimerization induced by the binding of VEGF (**Figure 1F**). The model includes a multi-compartmental receptor cycling model that comprises two compartments as signaling endosomes to more accurately fit the experimental data (**Figure 1F**). The transition from the signaling endosome to the recycling endosome is dependent on NRP1 consistent with experimental data (Ballmer-Hofer et al., 2011) and may be modified by TSP1. TSP1 may also affect the degradation of receptors from the signaling endosome as shown in the diagram (**Figure 1F**). Consistent with the experimental evidence, only unphosphorylated receptors cycle back to the plasma membrane from the recycling compartment (Ballmer-Hofer et al., 2011). The signaling pathway downstream of the activated VEGFR2 is presented in **Figure 2A**.

The signal transduction pathway is constructed by taking into account recent experimental evidence demonstrating that Axl-1 and Src are crucial for the transduction of the signal from phosphorylated receptor to Akt (Ruan and Kazlauskas, 2012a; Sun et al., 2012). We also reviewed the information available on PI3K/AKT pathway in the Reactome Pathway Database in order to ensure the consistency of the pathway structure with the available data (Milacic et al., 2012; Croft et al., 2014). TSAd and Src are lumped together (TSAd/Src) and activated (Src phosphorylation) as a function of phosphorylated VEGFR2 (pR2) presented in detailed in the Supplementary Materials (Sun et al., 2012; Gordon et al., 2016). Activated Src phosphorylates Axl-1 which then induces Axl-1 autophosphorylation on a different site consistent with experimental data (Ruan and Kazlauskas, 2012a,b). PI3K is then recruited and activated by Axl-1. Once in the membrane, PI3K phosphorylates PIP2 to generate PIP3. PIP3 recruits PDK1 and Akt to the membrane from the cytoplasm through their PH domains. The kinase mTOR phosphorylates Akt on serine 473 residue (S473) (Sarbassov et al., 2005) followed by the phosphorylation of the threonine 308 residue (T308) residue on Akt by PDK1 (Scheid et al., 2002).

A different arm of the pathway from the phosphorylated VEGFR2 is the activation of PLCγ (Meyer et al., 2003) which leads to the elevation of IP3 and Ca release from the ER via the IP3-sensitive receptors (**Figure 2B**). Ca activates CaM which then binds eNOS–caveolin-1 complex and results in the dissociation of caveolin-1 and partial activation of eNOS (Dudzinski and Michel, 2007; Balligand et al., 2009). Phosphorylation of eNOS on serine 1177 residue (S1177) by activated Akt is included as shown in **Figure 2A**. Phosphorylated eNOS is fully active and is shown to be insensitive to Ca (Dimmeler et al., 1999). The Ca module is similar to the previous model with Ca-induced Ca release channels (CRAC), ER release via IP3 receptors, and Ca pumps (Silva et al., 2007; Schmeitz et al., 2013; Bazzazi and Popel, 2017).

### Model Parameterization and Sensitivity Analysis

**FIGURE 3:**
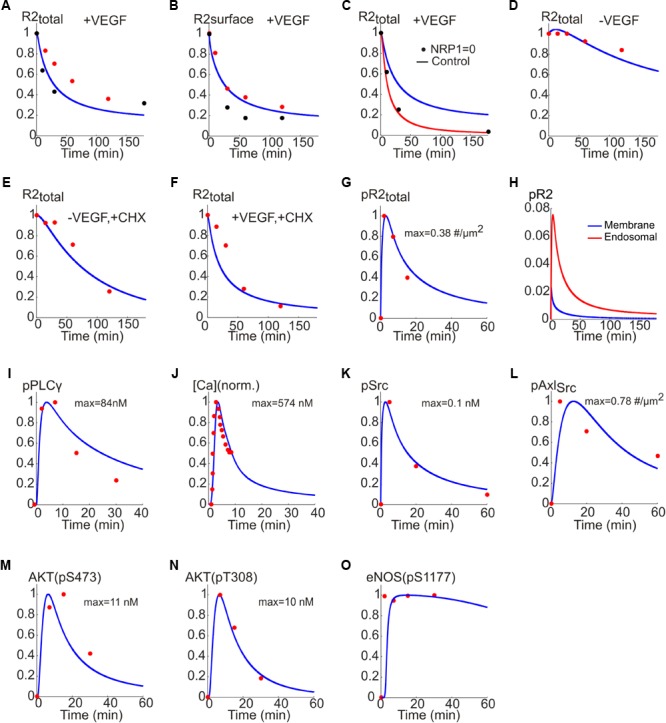
Fitting the model to experimental data. **(A)** Total receptor level versus time fitted to the data in Bruns et al. (2010) (red) and Ballmer-Hofer et al. (2011) (black). **(B)** Surface receptor versus time fitted to the data in Ewan et al. (2006) (red) and Bruns et al. (2010) (black). **(C)** Total receptor level in the presence (blue trace) and absence of NRP1 (red trace) fitted to the data in Ballmer-Hofer et al. (2011) (black). **(D)** Total receptor level under control condition. **(E)** Total receptor level in the presence of CHX and the absence of VEGF. **(F)** With VEGF and CHX. **(G)** Dynamics of pR2 traces versus time fitted to the data in Bruns et al. (2010) (red). **(H)** pR2 signaling from endosomal and membrane compartments. **(I)** PLCγ temporal dynamics fitted to the data in Chabot et al. (2009) (red). **(J)** Cytoplasmic Ca concentration fitted to the data in Li et al. (2011) (red). **(K)** Phosphorylated Src dynamics from the model fitted to the data in Ruan and Kazlauskas (2012a) (red). **(L)** Dynamics of Src-phosphorylated Axl-1 fitted to the data in Ruan and Kazlauskas (2012a) (red). **(M)** Dynamics of phosphorylated S473 on Akt fitted to the data in Ruan and Kazlauskas (2012a) (red). **(N)** Dynamics of phosphorylated T308 on Akt fitted to data in Ruan and Kazlauskas (2012a) (red). **(O)** Dynamics of phosphorylated S1177 on eNOS fitted to the data in Chabot et al. (2009) (red).

To estimate model parameters, we fit the model to a consistent set of experimental data from human endothelial cells (HUVEC) as shown in **Figure 3**. **Figures 3A,B** show the model fit to the data for the normalized total receptor dynamics (Bruns et al., 2010; Ballmer-Hofer et al., 2011) and surface receptor level time course (Ewan et al., 2006; Bruns et al., 2010). The initial receptor levels were selected based on the measured experimental data in Imoukhuede and Popel (2012, 2014). In the absence of NRP1, VEGFR2 degradation is enhanced and there is little transfer of the internalized receptors to the recycling endosome (Ballmer-Hofer et al., 2011).

The model correctly captures this as shown in **Figure 3C** (control trace with NRP1 is presented in blue versus simulation in red and experimental data shown as black circles). The model is also fitted to the data in the presence or absence of cycloheximide (CHX), a potent inhibitor of protein synthesis. This is to estimate the effective rate of protein synthesis in the model, namely the parameter ksingleR2_syn_. The model adequately fits the data under control condition (no VEGF, no CHX) as in **Figure 3D**. In the absence of CHX, the VEGFR2 levels drop considerably due to inhibition of receptor synthesis (**Figure 3E** versus **Figure 3D**). With both VEGF and CHX present, the drop in VEGFR2 levels is even faster, fitting the available experimental data (**Figure 3F**).

While constructing the model we determined that having two signaling internalized compartments (**Figure 1F**) was necessary for better fitting of the model to the set of data on receptor dynamics. The value of ksingleR2_syn_ is computed to be about 1.4e-4 s^-1^ (this translates into a synthesis rate of ∼0.84 receptor/s). The normalized phosphorylated VEGFR2 versus time is fitted to the data in Chabot et al. (2009) and shows the expected rapid transient increase in phosphorylated VEGFR2 that decreases to lower levels at the steady state (**Figure 3G**).

It is important to differentiate how much of the downstream signal comes from activated VEGFR2 on the membrane versus endosomes (**Figure 3H**). The model predicts that the majority of the signal comes from the internalized receptors (red line) while the signal from surface receptors is short lived (blue). Note that this is an emergent property of the model and is not an *a priori* assumption. In fact, this is consistent with recent experimental evidence demonstrating that in many receptor tyrosine kinases including VEGFR2 the bulk of signaling comes from the internalized receptors (Weddell and Imoukhuede, 2017).

To fit the Ca regulation arm of the pathway, phosphorylated PLCγ trace from the model is fitted to the experimental data in Chabot et al. (2009; **Figure 3I**). Additionally, the cytoplasmic Ca trace from the model and the data from Li et al. (2011) are fitted (**Figure 3J**) to constrain the parameters for the Ca cycling module. For the signaling to PI3K/AKT the data in Ruan and Kazlauskas (2012a) were utilized. Phosphorylated Src and the corresponding data points are shown in **Figure 3K**. Also shown in **Figure 3L**, is the Src phosphorylated Axl-1 dynamics fitted to the data points. The traces for the phosphorylation of S473 and T308 residues on Akt are shown in **Figures 3M,N**, respectively; the data points are taken from Chabot et al. (2009). The Akt-phosphorylated eNOS (on S1177) trace is shown in **Figure 3O** along with the experimental data from Chabot et al. (2009). Rich set of experimental data enabled us to achieve a good fit for the baseline model allowing further investigation of the therapeutic interventions on important pathway outputs (i.e., Akt and eNOS). The parameter values along with descriptions are given in Supplementary Table S1.

**FIGURE 4:**
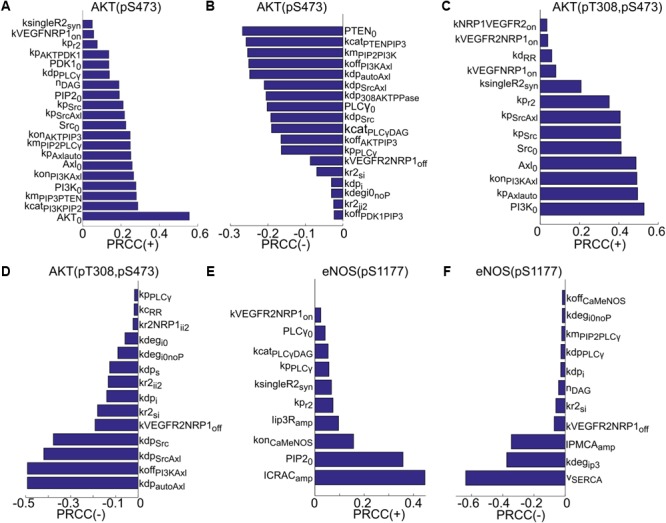
Global sensitivity analysis using PRCC. **(A)** Ranking of positively correlated parameters determining pS473 on Akt. **(B)** Ranking of negatively correlated parameters determining pS473. **(C)** Positively correlated parameters ranked in the order of sensitivity determining doubly phosphorylated Akt. **(D)** Negatively correlated parameters ranked in the order of sensitivity determining doubly phosphorylated Akt. **(E)** Ranking of positively correlated parameters determining pS1177. **(F)** Ranking of negatively correlated parameters.

Global sensitivity analysis is then carried out using the partial rank correlation coefficient (PRCC) method described in Marino et al. (2008) to identify the most sensitive parameters that determine the activation of Akt and eNOS. The results are summarized in **Figure 4**. Both positively and negatively correlated parameters are included. The sensitive positively and negatively correlated parameters determining the activation of S473 on Akt are shown in **Figures 4A,B**. The top positively correlated parameters (**Figure 4A**) are the catalytic rate of PI3K (kcat_PI3KPIP2_), PIP3 concentration for half activation of PTEN (km_PIP3PTEN_), the total level of PI3K (PI3K_0_), and several parameters determining activation of Axl-1 (kon_PI3KAxl_, Axl_0_, kp_Axlauto_, kp_SrcAxl_). Notably, synthesis rate of VEGFR2 (ksingleR2_syn_) and the on-rate for the binding of VEGF to NRP1 (kVEGFNRP1_on_) are also significant parameters. The top negatively correlated parameters include the total level of PTEN (PTEN_0_), the catalytic rate of PTEN (kcat_PTENPIP3_), the PIP3 concentration for half-maximal activation of PI3K (km_PIP2PI3K_), the inactivation rate of PI3K (koff_PI3KAxl_), and the dephosphorylation rate of Axl-1 (kdp_autoAxl_ and kdp_SrcAxl_). Other notable parameters include the catalytic rate of PLCγ (kcat_PLCγDAG_), the off-rate of VEGF-VEGFR2 unbinding from NRP1 (kVEGFR2NRP1_off_), and the internalization rate of VEGFR2 in the absence of NRP1 (kr2_si_).

For the doubly phosphorylated Akt (phosphorylated on both S473 and T308), the top positively correlated parameters are the total level of PI3K, the autophosphorylation rate of Axl-1 (kp_Axlauto_), and the rate of PI3K activation by Axl-1 (kon_PI3KAxl_). The total level of Axl-1 (Axl_0_) and Src (Src_0_) and the phosphorylation rate of VEGFR2 are significant. Other notable parameters are VEGFR2 synthesis rate, the on-rate of VEGF binding to NRP1 (kVEGFNRP1_on_), and ligand-independent decoupling rate between receptors (kd_RR_).

The top negatively correlated parameters are the dephosphorylation rate of autophosphorylated Axl-1, the rate of PI3K inactivation, and the dephosphorylation rate of the site on Axl-1 phosphorylated by Src. Other notable parameters include the dephosphorylation rate of VEGFR2 (kdp_s_ and kdp_i_), and the degradation rate of the receptor (kdeg_i0noP_ and kdeg_i0_). The top positively correlated parameters determining phosphorylated eNOS on S1177 are the amplitude of the CRAC channel current (ICRAC_amp_), the total level of PIP2 (PIP2_0_), the on-rate of Ca/CaM binding to eNOS–caveolin complex, and the amplitude of current through the IP3-sensitive receptors (Iip3R_amp_). The synthesis rate of VEGFR2 and catalytic rate of PLCγ are also notable positively correlated parameters. Top negatively correlated parameters are the current through the SERCA pump (v_SERCA_), the degradation rate of IP3 (kdeg_ip3_), and the plasma membrane Ca pump rate (IPMCA_amp_).

Sensitivity analysis provides general understanding of the mechanisms that determine the activation of Akt and eNOS and specifies the rate limiting factors within the context of this model in the presence of VEGF. We next apply the model to test potential mechanisms for the inhibition of signaling to Akt and eNOS by TSP1.

### Enhanced Degradation of VEGFR2 From the Signaling Endosomes by TSP1 Is Sufficient to Explain the Inhibition of eNOS Activation by TSP1

There is experimental evidence that TSP1 binding to CD47 inhibits VEGFR2, Akt, and Src activation (Kaur et al., 2010, 2014). There is also limited experimental evidence that TSP1 accelerates the degradation of VEGFR2 (Kaur et al., 2011). In a previous modeling investigation, this enhanced degradation by TSP1 was sufficient to explain the inhibition of VEGFR2 activation and downstream signaling (Bazzazi et al., 2017). In this study, we aimed at utilizing our detailed multi-compartmental model of receptor cycling to distinguish between two competing mechanisms for enhancing the degradation of VEGFR2 by TSP1. For the simulations in this section, 2.2 nM TSP1 is added for 10 min before addition 50 ng/ml VEGF similar to the experimental protocol (Kaur et al., 2010).

**FIGURE 5:**
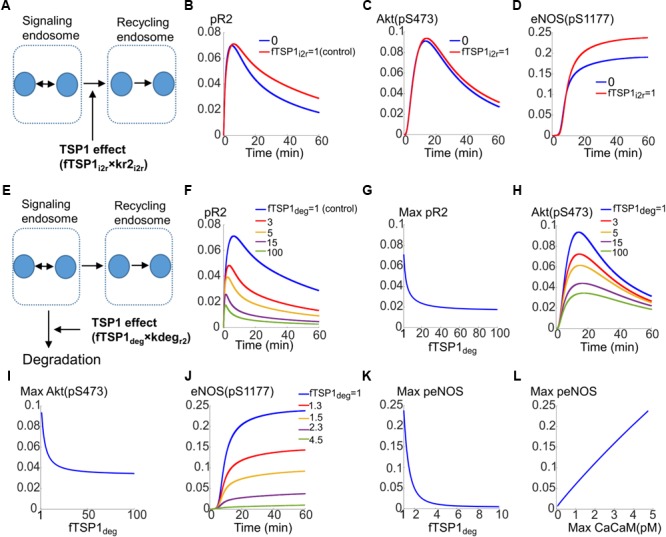
Mechanisms for the inhibition of VEGFR2 signaling to Akt and eNOS by TSP1. **(A)** Potential mechanism for TSP1 inhibition of VEGFR2 activation involving the blockade of receptor trafficking from signaling endosomes to recycling endosomes. **(B)** pR2 temporal dynamics under control (red) and complete trafficking inhibition conditions (blue). **(C)** Akt dynamics of pS473. **(D)** Dynamics of eNOS phosphorylation on S1177. **(E)** Mechanisms of TSP1 inhibition involving enhanced degradation of VEGFR2 from the signaling endosomes. **(F)** Traces of pR2 in response to changes in TSP1-mediated degradation parameter. **(G)** Maximum pR2 versus the degradation parameter. **(H)** Traces illustrating the dynamics of pS473 on Akt in response to changes in enhanced degradation parameter. **(I)** Maximum pS473 on Akt versus enhanced degradation parameter. **(J)** Dynamics of pS1177 on eNOS as a response to changes in receptor degradation. **(K)** Maximum pS1177 on eNOS as a function of enhanced degradation of receptors indicating sensitive dependence on TSP1-mediated enhancement of degradation. **(L)** Maximum peNOS versus maximum Ca/CaM illustrating a near-perfect linear relation.

The first possibility is that TSP1 inhibits the transport of receptors from the signaling endosomes to the recycling endosome as illustrated in **Figure 5A**. The effect is modeled by the parameter fTSP1_i2r_ which takes values between 0 (total inhibition of transport) and 1 (control). As demonstrated by the simulations in **Figures 5B–D**, total block of receptor transport from signaling endosomes to the recycling endosomes only slightly reduces VEGFR2 phosphorylation (**Figure 5B**), Akt activation (**Figure 5C**), or eNOS phosphorylation (**Figure 5D**).

The second mechanism for TSP1 inhibition of VEGFR2 signaling considered here is that TSP1 enhances degradation from the signaling endosomes (**Figure 5E**, also see **Figure 1F**). This is modeled by the parameter fTSP1_deg_ (equals 1 for control, >1 when simulating enhanced degradation). As shown in **Figure 5F**, this mechanism is sufficient to explain inhibition of VEGFR2 phosphorylation as a fivefold increase in degradation represses pR2. **Figure 5G** illustrates the changes in maximum pR2 as a function of enhanced degradation. Increasing degradation ∼20-fold is predicted to be sufficient to lead to near maximal inhibition. The degradation from signaling endosomes also reduces Akt activation as shown in **Figure 5H**, however, the inhibition plateaus (**Figure 5I**).

Current mechanism, therefore, does not explain full inhibition of Akt phosphorylation on S473 observed experimentally (Kaur et al., 2010). This suggests that there is sufficient signaling from the surface receptors to activate Akt on S473. This implies that there are other mechanisms at play that contribute to full inhibition of Akt activation.

However, activation of eNOS is potently inhibited by the proposed mechanism as shown in **Figures 5J,K**. A fourfold increase in degradation is sufficient to abolish eNOS activation (**Figure 5J**). Interestingly, max peNOS is linearly correlated with max CaCaM (**Figure 5L**) predicting that the inhibition is mainly through the inhibition of Ca/CaM. In other words, TSP1 effect on VEGFR2 potently inhibits Ca/CaM elevation downstream of VEGFR2 thereby blocking the initial activation of eNOS by Ca/CaM, the necessary step in the activation of eNOS (Dudzinski and Michel, 2007; Balligand et al., 2009).

### Phosphatase Recruitment to VEGFR2 Is Necessary to Explain the Complete Inhibition of Akt Phosphorylation on S473 by TSP1

Simulations in the previous section indicated that enhanced degradation of receptors by TSP1 from signaling endosomes was not sufficient in itself to explain Akt inhibition of phosphorylation on S473. We thus postulated that phosphatase recruitment to the receptor by TSP1–CD47 interaction might explain full inhibition of Akt and in concert with enhanced degradation mechanism explain the full range of TSP1 inhibitory effects. Phosphatase recruitment to CD47 by TSP1 has been observed experimentally and might support the proposal here (Yao et al., 2014).

**FIGURE 6:**
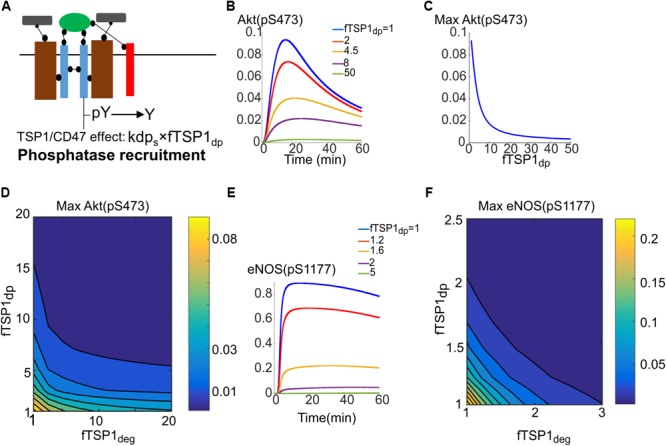
Phosphatase recruitment by TSP1 is required for complete inhibition of Akt phosphorylation on S473. **(A)** Diagram illustrating phosphatase recruitment to the receptor by TSP1-CD47. **(B)** Akt activation is efficiently inhibited by changes in membrane dephosphorylation rate mediated by TSP1. **(C)** Maximum Akt phosphorylation on S473 versus phosphatase recruitment parameter. **(D)** Two-parameter scan for maximum pS473 when both enhanced degradation and phosphatase recruitment are involved. **(E)** Sensitive dependence of eNOS phosphorylation to increases in membrane level dephosphorylation by TSP1. **(F)** Two-parameter scan for pS1177 on eNOS in response to TSP1-mediated changes in enhanced degradation and phosphatase recruitment.

The simulations should be viewed as test of hypothesis that should motivate and guide additional experimental investigation. The proposed mechanism is illustrated in **Figure 6A** and modeled by parameter fTSP1_dp_ which describes potential increase in phosphatase recruitment to surface receptors (control value of 1). Traces in **Figure 6B** demonstrate the effectiveness of this mechanism in inhibiting Akt activation. **Figure 6C** further shows that 20-fold increase in dephosphorylation rate potently inhibits Akt activation. In reality, it is plausible for both enhanced degradation and phosphatase recruitment mechanisms to work in concert as shown in **Figure 6D** that shows the effect of changing both parameters on Akt activation. What the simulation shows is that a 10-fold increase in dephosphorylation and degradation each would be sufficient to explain TSP1 inhibition. Phosphatase recruitment would also augment enhanced degradation in the inhibition of eNOS activation as shown in **Figures 6E,F**. What these simulations show is that when both mechanisms work in concert, the inhibitory effect of TSP1 may be very potent.

In the next section, we utilize this knowledge to perform simulations for the inhibition of TSP1–CD47 signaling as pro-angiogenic intervention.

### Inhibition of TSP1–CD47 Axis to Rescue VEGF Signaling

**FIGURE 7:**
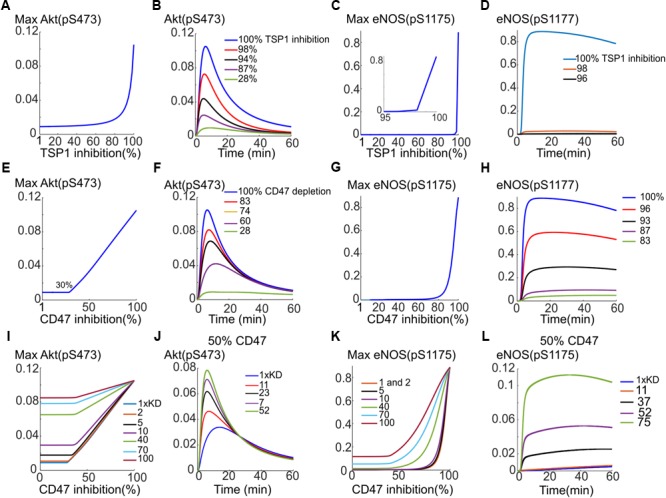
Rescuing VEGF signaling to Akt and eNOS by targeting TSP1 or CD47. **(A)** Depletion of TSP1 and the predicted recovery of pS473 on Akt. **(B)** Sample traces for pS473 for different values of TSP1 inhibition. **(C)** Recovery of max pS1177 on eNOS in response to TSP1 inhibition. **(D)** Sample traces for phosphorylated eNOS. **(E)** Rescue of Akt phosphorylation on S473 with depletion of CD47 indicating a linear recovery. **(F)** Sample traces for pS473 for different depletion levels. **(G)** pS1177 in response to CD47 depletion. **(H)** Sample pS1177 traces requiring over 90% depletion for sufficient recovery of the activation signal. **(I)** Combining CD47 depletion with the inhibition of TSP1 binding to CD47 achieved by increasing the Kd of TSP1-CD47 binding. Akt activation recovery for different values of increased Kd relative to control (blue, the lowest curve). **(J)** Restoring Akt signaling when 50% CD47 depletion is combined with TSP1-CD47 binding inhibition. **(K)** Improved recovery of maximum pS1177 on eNOS under combination intervention. **(L)** The temporal recovery traces for pS1177 when 50% depletion in CD47 is combined with inhibition of TSP1 binding to CD47 as shown by the increase in Kd.

Previous simulations illustrate that TSP1–CD47 inhibition is very effective in shutting down VEGF signaling to Akt and eNOS via enhancing the degradation of VEGFR2 from signaling endosome coupled with phosphatase recruitment to the plasma membrane. Simulations indicated that a 10-fold increase in both phosphatase recruitment and VEGFR2 degradation would effectively block both Akt and eNOS activation. Here we aim at utilizing the model to test different interventions to best rescue VEGF signaling to Akt and eNOS in the presence of TSP1. This may be relevant to disease conditions such as wound healing and PAD where angiogenesis mediated by VEGF may be compromised. We initially simulate the effect of depleting TSP1 in the extracellular space; for example, under *in vivo* conditions such depletion could be achieved by administration of a TSP1-specific antibody. As shown in **Figures 7A,B**, 80% depletion of TSP1 is necessary for the Akt activation signal to begin to recover (**Figure 7A**) and for effective rescue of Akt signal, a 98% inhibition is necessary (**Figure 7B**). However, this is unlikely to be achievable physiologically. The rescue of eNOS activation with this intervention is even less efficient as shown in **Figures 7C,D**. We next postulate that depleting CD47, the receptor for TSP1, might be more efficient in the restoration of VEGF signaling to Akt and eNOS. Indeed, Akt activation is restored in a linear fashion after ∼30% of CD47 were depleted (**Figure 7E**). The reason for the initial delay is that assumed total CD47 level of 10,000 receptors is higher than the total number of VEGFR2 at 6000. Because VEGFR2 and CD47 are constitutively associated with each other, initial depletion is necessary to reduce the number of CD47 to a comparable level as VEGFR2. The traces in **Figure 7F** show the gradual restoration in Akt activation.

While eNOS activation in response to CD47 inhibition is less steep than the case of TSP1 depletion (**Figure 7G** versus **Figure 7C**), the simulations still predict difficulty in the plausibility of targeting CD47 to rescue eNOS. The recovery curve still has a strong switching behavior with minimum 80% depletion necessary for peNOS recovery. The traces for peNOS in **Figure 7H** also indicate that 96% inhibition may be required to sufficiently restore eNOS activation.

The model predicts that neither CD47 nor TSP1 depletion is efficient in eNOS activation rescue. We therefore hypothesized that combining CD47 depletion with a hypothetical therapy that inhibits TSP1 binding to CD47 (modeled by increasing the Kd of TSP1 binding to CD47) may produce a better recovery curve for both Akt and eNOS. Indeed, simulations predict that a hypothetical therapy, such as a TSP1-specific antibody, that sequesters TSP1 and reduces TSP1 binding to CD47 by 10-fold (increasing Kd by 10-fold), improves the anti-CD47 recovery curve for Akt (**Figure 7I**, the blue trace for control Kd versus the purple trace with 10× Kd). Considering a different scenario, suppose CD47 is depleted by 50%, then a 23-fold reduction in TSP1 binding to CD47 would result in near complete recovery of Akt (**Figure 7J**, black trace). Similarly, the recovery in maximum eNOS is much improved if both interventions are combined as in **Figure 7K**. As illustrated in **Figure 7K**, a 70-fold decrease in the binding of TSP1 to CD47 (a 70-fold increase in Kd) by a hypothetical compound would significantly improve the recovery of eNOS activation in response to CD47 depletion (**Figure 7K**, blue trace).

Suppose again that 50% of CD47 has been depleted, a 52-fold reduction in the binding of TSP1 to CD47 would lead to a significant restoration of eNOS activation signal (**Figure 7L**, purple curve). Simulations here predict that while Akt activation may be rescued efficiently by targeting CD47 alone (rather than TSP1), to effectively restore eNOS activation, combination therapies that target both CD47 and another mechanism such as the binding of TSP1 to CD47 may be necessary. Overall, computational modeling suggests that targeting TSP1–CD47 axis has potential as a pro-angiogenic intervention. Modeling results also suggest that inhibiting CD47 might be more advantageous than TSP1 alone. However, a dual approach that captures both TSP1 and CD47 is predicted to be optimal.

## Discussion

A detailed model of VEGFR2 signaling to Akt and eNOS was constructed in order to test precise mechanisms of the experimentally observed inhibition of this pathway by TSP1 (Kaur et al., 2010, 2014). Empirically, TSP1 has been found to affect VEGFR2 trafficking and degradation (Kaur et al., 2011). Considering the available evidence, we tested two different nodes that might be targets for TSP1 effects. TSP1 may inhibit VEGFR2 transport from signaling endosomes to recycling endosomes, hence, preventing the recycling of the receptors to the membrane. Interestingly, the model predicted that this mechanism did not lead to the inhibition of VEGFR2 phosphorylation, Akt activation, and eNOS phosphorylation on S1177. This is consistent with experimental data showing that siRNA knockdown of NRP1 does not abolish VEGFR2 activation, and that cell migration is compromised via the inhibition of focal adhesion kinases. The model, however, showed that enhancing receptor degradation from early endosomes can explain the inhibition of VEGFR2 and eNOS activation, and partially the inhibition of Akt phosphorylation on S473. This implied that signaling from membrane-bound receptors is sufficient to sustain Akt signaling. We thus hypothesized that phosphatase recruitment by TSP1–CD47 may explain the complete inhibition of Akt phosphorylation on S473 observed experimentally. The phosphatase recruitment hypothesis was indeed predicted to be very effective in shutting down Akt activation.

An interesting outcome of the simulations presented here is that TSP1 is particularly effective in inhibition of eNOS and that eNOS activation is very sensitive to perturbations introduced by TSP1–CD47. This may provide a mechanistic explanation for potent inhibition of NO signaling by TSP1. Simulations further suggest that the main mechanism for the sensitive inhibition of eNOS activation is through inhibition of Ca/CaM. The effect on Ca/CaM signaling by TSP1 has not been explored in detail within current experimental literature. Our computational model suggests the need for further investigations in this area as we predict a potent inhibition of Ca/CaM signaling by TSP1 that may go beyond eNOS and influence other Ca/CaM targets in endothelial as well as other cell types. As Ca/CaM signaling plays critical roles in VEGF-induced angiogenesis (Shen et al., 2007; Jung et al., 2010; Banumathi et al., 2011), its potent inhibition by TSP1–CD47 may be another hallmark of TSP1 signaling.

Given the versatility of TSP1–CD47 interaction in blocking angiogenesis, it is plausible to explore the possibility of inhibiting TSP1, CD47, or both together as strategies to enhance or rescue angiogenesis. Blocking TSP1 and CD47 has already been explored in different setting. For example, inhibiting CD47 alleviates pathogenic effects of aging, a process that may be associated with deterioration in angiogenic capacity, on different tissues in response to ischemia (Isenberg et al., 2007a, 2008a,b). Utilizing our model, we simulated different strategies for the inhibition of TSP1–CD47 signaling to evaluate the recovery response of Akt and eNOS. Our simulations predict that depleting CD47 is more advantageous in rescuing Akt signal in response to VEGF; however, with regards to eNOS activation, our model predicts that combination therapy combining CD47 depletion with the inhibition of TSP1 binding to CD47 (hypothetical compound that effectively increases the dissociation constant) may be necessary for effective rescue. Given the highly non-linear nature of these signaling pathways, these non-trivial findings would have been difficult to hypothesize without the application of mechanistic modeling.

### Model Limitations

Thrombospondin-1 engages other receptors that could play a role in angiogenesis and these should be tested in future studies. Another limiting factor in all these predictions is that the model only considers VEGF signaling through VEGFR2 to Akt and eNOS and ignores other sources of eNOS activation in tissues. Moreover, the model ignores the pro-apoptotic signaling downstream of TSP1–CD47 that might override other angio-inhibitory signaling (Rath et al., 2006; Kaur et al., 2013; Gao et al., 2016).

Thrombospondin-1 via CD47 can also adversely impact cellular metabolism under some conditions (Miller et al., 2015). Thus, the pro-survival action of TSP1–CD47 inhibition might be a more significant outcome than rescuing signals through VEGF. The computational model may be enhanced by incorporating mechanisms that control TSP1 expression [such as miroRNAs as modeled in Zhao et al. (2017)] which would enable investigation of different targeting strategies in combination with what we have already included here. However, in the absence of more clarifying data, our proof-of-concept simulations point at clear distinctions between multiple strategies for rescuing VEGF signaling by blocking TSP1–CD47 axis.

To summarize, the computational investigation here synthesizes available knowledge about VEGF signaling and TSP1–CD47 to propose molecular mechanisms of the inhibition of the VEGF pathway by TSP1. The mechanistic model also paves the way for *in silico* investigation of therapeutic strategies for promoting angiogenesis that may prove relevant for diseases such as PAD and other age-related conditions with no currently available effective therapies.

## Author Contributions

HB and AP conceived and designed the study. JI and BA provided critical input into the study. HB implemented the model in BioNetGen, performed the computer simulations, analyzed the data, and wrote the manuscript. JI, BA, YZ, MJ, and AP participated in writing and editing the manuscript.

## Conflict of Interest Statement

JI serves as Science Advisory Board Chair of Radiation Control Technologies, Inc. (Jersey City, NJ, United States) and has equity interests in the same and Tioma Therapeutics (St. Louis, MO, United States) which have licensed CD47 technologies. The other authors declare that the research was conducted in the absence of any commercial or financial relationships that could be construed as a potential conflict of interest.

## References

[B1] Annex B. H. (2013). Therapeutic angiogenesis for critical limb ischaemia. *Nat. Rev. Cardiol.* 10 387–396. 10.1038/nrcardio.2013.70 23670612

[B2] Bagavandoss P. Wilks J. W. (1990). Specific inhibition of endothelial cell proliferation by thrombospondin. *Biochem. Biophys. Res. Commun.* 170 867–872. 10.1016/0006-291X(90)92171-U 1696478

[B3] Balligand J. L. Feron O. Dessy C. (2009). eNOS activation by physical forces: from short-term regulation of contraction to chronic remodeling of cardiovascular tissues. *Physiol. Rev.* 89 481–534. 10.1152/physrev.00042.2007 19342613

[B4] Ballmer-Hofer K. Andersson A. E. Ratcliffe L. E. Berger P. (2011). Neuropilin-1 promotes VEGFR-2 trafficking through Rab11 vesicles thereby specifying signal output. *Blood* 118 816–826. 10.1182/blood-2011-01-328773 21586748

[B5] Banumathi E. O’connor A. Gurunathan S. Simpson D. A. Mcgeown J. G. Curtis T. M. (2011). VEGF-induced retinal angiogenic signaling is critically dependent on Ca^2+^ signaling by Ca^2+^/calmodulin-dependent protein kinase II. *Invest. Ophthalmol. Vis. Sci.* 52 3103–3111. 10.1167/iovs.10-6574 21310919

[B6] Bao P. Kodra A. Tomic-Canic M. Golinko M. S. Ehrlich H. P. Brem H. (2009). The role of vascular endothelial growth factor in wound healing. *J. Surg. Res.* 153 347–358. 10.1016/j.jss.2008.04.023 19027922 PMC2728016

[B7] Bauer E. M. Qin Y. Miller T. W. Bandle R. W. Csanyi G. Pagano P. J. (2010). Thrombospondin-1 supports blood pressure by limiting eNOS activation and endothelial-dependent vasorelaxation. *Cardiovasc. Res.* 88 471–481. 10.1093/cvr/cvq218 20610415 PMC2972685

[B8] Bazzazi H. Isenberg J. Popel A. (2017). Inhibition of VEGFR2 activation and its downstream signaling to ERK1/2 and calcium by Thrombospondin-1 (TSP1): *In silico* investigation. *Front. Physiol.* 8:48 10.3389/fphys.2017.00048 PMC5292565 28220078

[B9] Bazzazi H. Popel A. S. (2017). Computational investigation of sphingosine kinase 1 (SphK1) and calcium dependent ERK1/2 activation downstream of VEGFR2 in endothelial cells. *PLoS Comput. Biol.* 13:e1005332. 10.1371/journal.pcbi.1005332 28178265 PMC5298229

[B10] Boucher J. M. Bautch V. L. (2014). Antiangiogenic VEGF-A in peripheral artery disease. *Nat. Med.* 20 1383–1385. 10.1038/nm.3767 25473918

[B11] Bruns A. F. Herbert S. P. Odell A. F. Jopling H. M. Hooper N. M. Zachary I. C. (2010). Ligand-stimulated VEGFR2 signaling is regulated by co-ordinated trafficking and proteolysis. *Traffic* 11 161–174. 10.1111/j.1600-0854.2009.01001.x 19883397

[B12] Carmeliet P. Ferreira V. Breier G. Pollefeyt S. Kieckens L. Gertsenstein M. (1996). Abnormal blood vessel development and lethality in embryos lacking a single VEGF allele. *Nature* 380 435–439. 10.1038/380435a0 8602241

[B13] Chabot C. Spring K. Gratton J. P. Elchebly M. Royal I. (2009). New role for the protein tyrosine phosphatase DEP-1 in Akt activation and endothelial cell survival. *Mol. Cell. Biol.* 29 241–253. 10.1128/MCB.01374-08 18936167 PMC2612487

[B14] Chen Y. Medhora M. Falck J. R. Pritchard K. A. Jr. Jacobs E. R. (2006). Mechanisms of activation of eNOS by 20-HETE and VEGF in bovine pulmonary artery endothelial cells. *Am. J. Physiol. Lung. Cell Mol. Physiol.* 291 L378–L385. 10.1152/ajplung.00424.2005 16679377

[B15] Claesson-Welsh L. Welsh M. (2013). VEGFA and tumour angiogenesis. *J. Intern. Med.* 273 114–127. 10.1111/joim.12019 23216836

[B16] Clegg L. E. Ganta V. C. Annex B. H. Mac Gabhann F. (2017). Systems pharmacology of VEGF165b in peripheral artery disease. *CPT Pharmacometrics Syst. Pharmacol.* 6 833–844. 10.1002/psp4.12261 29193887 PMC5744173

[B17] Clegg L. E. Mac Gabhann F. (2018). A computational analysis of pro-angiogenic therapies for peripheral artery disease. *Integr. Biol.* 10 18–33. 10.1039/C7IB00218A 29327758 PMC7017937

[B18] Clegg L. W. Mac Gabhann F. (2015). Site-specific phosphorylation of VEGFR2 is mediated by receptor trafficking: insights from a computational model. *PLoS Comput. Biol.* 11:e1004158. 10.1371/journal.pcbi.1004158 26067165 PMC4466579

[B19] Croft D. Mundo A. F. Haw R. Milacic M. Weiser J. Wu G. (2014). The reactome pathway knowledgebase. *Nucleic Acids Res.* 42 D472–D477. 10.1093/nar/gkt1102 24243840 PMC3965010

[B20] Curwen J. O. Musgrove H. L. Kendrew J. Richmond G. H. Ogilvie D. J. Wedge S. R. (2008). Inhibition of vascular endothelial growth factor-a signaling induces hypertension: examining the effect of cediranib (recentin; AZD2171) treatment on blood pressure in rat and the use of concomitant antihypertensive therapy. *Clin. Cancer Res.* 14 3124–3131. 10.1158/1078-0432.CCR-07-4783 18483380

[B21] Dellinger M. T. Brekken R. A. (2011). Phosphorylation of Akt and ERK1/2 is required for VEGF-A/VEGFR2-induced proliferation and migration of lymphatic endothelium. *PLoS One* 6:e28947. 10.1371/journal.pone.0028947 22174934 PMC3236226

[B22] Dimmeler S. Fleming I. Fisslthaler B. Hermann C. Busse R. Zeiher A. M. (1999). Activation of nitric oxide synthase in endothelial cells by Akt-dependent phosphorylation. *Nature* 399 601–605. 10.1038/21224 10376603

[B23] Dudzinski D. M. Michel T. (2007). Life history of eNOS: partners and pathways. *Cardiovasc. Res.* 75 247–260. 10.1016/j.cardiores.2007.03.023 17466957 PMC2682334

[B24] Ewan L. C. Jopling H. M. Jia H. Mittar S. Bagherzadeh A. Howell G. J. (2006). Intrinsic tyrosine kinase activity is required for vascular endothelial growth factor receptor 2 ubiquitination, sorting and degradation in endothelial cells. *Traffic* 7 1270–1282. 10.1111/j.1600-0854.2006.00462.x 17004325

[B25] Faeder J. R. Blinov M. L. Hlavacek W. S. (2009). Rule-based modeling of biochemical systems with BioNetGen. *Methods Mol. Biol.* 500 113–167. 10.1007/978-1-59745-525-1_5 19399430

[B26] Feliers D. Chen X. Akis N. Choudhury G. G. Madaio M. Kasinath B. S. (2005). VEGF regulation of endothelial nitric oxide synthase in glomerular endothelial cells. *Kidney Int.* 68 1648–1659. 10.1111/j.1523-1755.2005.00575.x 16164642

[B27] Ferrara N. (2010). Vascular endothelial growth factor and age-related macular degeneration: from basic science to therapy. *Nat. Med.* 16 1107–1111. 10.1038/nm1010-1107 20930754

[B28] Folkman J. (2004). Endogenous angiogenesis inhibitors. *APMIS* 112 496–507. 10.1111/j.1600-0463.2004.apm11207-0809.x 15563312

[B29] Fuh G. Garcia K. C. De Vos A. M. (2000). The interaction of neuropilin-1 with vascular endothelial growth factor and its receptor flt-1. *J. Biol. Chem.* 275 26690–26695. 10.1074/jbc.M003955200 10842181

[B30] Fukumura D. Gohongi T. Kadambi A. Izumi Y. Ang J. Yun C. O. (2001). Predominant role of endothelial nitric oxide synthase in vascular endothelial growth factor-induced angiogenesis and vascular permeability. *Proc. Natl. Acad. Sci. U.S.A.* 98 2604–2609. 10.1073/pnas.041359198 11226286 PMC30185

[B31] Gao Q. Chen K. Gao L. Zheng Y. Yang Y. G. (2016). Thrombospondin-1 signaling through CD47 inhibits cell cycle progression and induces senescence in endothelial cells. *Cell Death Dis.* 7:e2368. 10.1038/cddis.2016.155 27607583 PMC5059850

[B32] Good D. J. Polverini P. J. Rastinejad F. Le Beau M. M. Lemons R. S. Frazier W. A. (1990). A tumor suppressor-dependent inhibitor of angiogenesis is immunologically and functionally indistinguishable from a fragment of thrombospondin. *Proc. Natl. Acad. Sci. U.S.A.* 87 6624–6628. 10.1073/pnas.87.17.6624 1697685 PMC54589

[B33] Gordon E. J. Fukuhara D. Westrom S. Padhan N. Sjostrom E. O. Van Meeteren L. (2016). The endothelial adaptor molecule TSAd is required for VEGF-induced angiogenic sprouting through junctional c-Src activation. *Sci. Signal.* 9:ra72. 10.1126/scisignal.aad9256 27436360

[B34] Greenaway J. Lawler J. Moorehead R. Bornstein P. Lamarre J. Petrik J. (2007). Thrombospondin-1 inhibits VEGF levels in the ovary directly by binding and internalization via the low density lipoprotein receptor-related protein-1 (LRP-1). *J. Cell. Physiol.* 210 807–818. 10.1002/jcp.20904 17154366 PMC3412056

[B35] Gupta K. Gupta P. Wild R. Ramakrishnan S. Hebbel R. P. (1999). Binding and displacement of vascular endothelial growth factor (VEGF) by thrombospondin: effect on human microvascular endothelial cell proliferation and angiogenesis. *Angiogenesis* 3 147–158. 10.1023/A:1009018702832 14517432

[B36] Hindmarsh A. C. Brown P. N. Grant K. E. Lee S. L. Serban R. Shumaker D. E. (2005). SUNDIALS: suite of nonlinear and differential/algebraic equation solvers. *ACM Trans. Math. Softw.* 31 363–396. 10.1145/1089014.1089020

[B37] Hlavacek W. S. Faeder J. R. Blinov M. L. Posner R. G. Hucka M. Fontana W. (2006). Rules for modeling signal-transduction systems. *Sci STKE* 2006:re6. 10.1126/stke.3442006re6 16849649

[B38] Imoukhuede P. I. Popel A. S. (2012). Expression of VEGF receptors on endothelial cells in mouse skeletal muscle. *PLoS One* 7:e44791. 10.1371/journal.pone.0044791 22984559 PMC3440347

[B39] Imoukhuede P. I. Popel A. S. (2014). Quantitative fluorescent profiling of VEGFRs reveals tumor cell and endothelial cell heterogeneity in breast cancer xenografts. *Cancer Med.* 3 225–244. 10.1002/cam4.188 24449499 PMC3987073

[B40] Isenberg J. S. Hyodo F. Pappan L. K. Abu-Asab M. Tsokos M. Krishna M. C. (2007a). Blocking thrombospondin-1/CD47 signaling alleviates deleterious effects of aging on tissue responses to ischemia. *Arterioscler. Thromb. Vasc. Biol.* 27 2582–2588. 10.1161/ATVBAHA.107.155390 17916772

[B41] Isenberg J. S. Jia Y. Fukuyama J. Switzer C. H. Wink D. A. Roberts D. D. (2007b). Thrombospondin-1 inhibits nitric oxide signaling via CD36 by inhibiting myristic acid uptake. *J. Biol. Chem.* 282 15404–15415. 10.1074/jbc.M701638200 17416590 PMC2430148

[B42] Isenberg J. S. Maxhimer J. B. Powers P. Tsokos M. Frazier W. A. Roberts D. D. (2008a). Treatment of liver ischemia-reperfusion injury by limiting thrombospondin-1/CD47 signaling. *Surgery* 144 752–761. 10.1016/j.surg.2008.07.009 19081017 PMC2635486

[B43] Isenberg J. S. Pappan L. K. Romeo M. J. Abu-Asab M. Tsokos M. Wink D. A. (2008b). Blockade of thrombospondin-1-CD47 interactions prevents necrosis of full thickness skin grafts. *Ann. Surg.* 247 180–190. 10.1097/SLA.0b013e31815685dc 18156939 PMC2432017

[B44] Isenberg J. S. Ridnour L. A. Dimitry J. Frazier W. A. Wink D. A. Roberts D. D. (2006). CD47 is necessary for inhibition of nitric oxide-stimulated vascular cell responses by thrombospondin-1. *J. Biol. Chem.* 281 26069–26080. 10.1074/jbc.M605040200 16835222

[B45] Isenberg J. S. Romeo M. J. Yu C. Yu C. K. Nghiem K. Monsale J. (2008c). Thrombospondin-1 stimulates platelet aggregation by blocking the antithrombotic activity of nitric oxide/cGMP signaling. *Blood* 111 613–623. 10.1182/blood-2007-06-098392 17890448 PMC2200855

[B46] Jung H. J. Kim J. H. Shim J. S. Kwon H. J. (2010). A novel Ca^2+^/calmodulin antagonist HBC inhibits angiogenesis and down-regulates hypoxia-inducible factor. *J. Biol. Chem.* 285 25867–25874. 10.1074/jbc.M110.135632 20554536 PMC2919149

[B47] Kaur S. Chang T. Singh S. P. Lim L. Mannan P. Garfield S. H. (2014). CD47 signaling regulates the immunosuppressive activity of VEGF in T cells. *J. Immunol.* 193 3914–3924. 10.4049/jimmunol.1303116 25200950 PMC4185246

[B48] Kaur S. Martin-Manso G. Pendrak M. L. Garfield S. H. Isenberg J. S. Roberts D. D. (2010). Thrombospondin-1 inhibits VEGF receptor-2 signaling by disrupting its association with CD47. *J. Biol. Chem.* 285 38923–38932. 10.1074/jbc.M110.172304 20923780 PMC2998110

[B49] Kaur S. Pendrak M. L. Garfield S. H. Roberts D. D. (2011). Thrombospndin 1 accelerates VEGFR2 trafficking and directs towards lysosomes for degradation. *FASEB J.* 25:1091.1010.

[B50] Kaur S. Soto-Pantoja D. R. Stein E. V. Liu C. Elkahloun A. G. Pendrak M. L. (2013). Thrombospondin-1 signaling through CD47 inhibits self-renewal by regulating c-Myc and other stem cell transcription factors. *Sci. Rep.* 3:1673. 10.1038/srep01673 23591719 PMC3628113

[B51] Kieran M. W. Kalluri R. Cho Y. J. (2012). The VEGF pathway in cancer and disease: responses, resistance, and the path forward. *Cold Spring Harb. Perspect. Med.* 2:a006593. 10.1101/cshperspect.a006593 23209176 PMC3543071

[B52] Ku D. D. Zaleski J. K. Liu S. Brock T. A. (1993). Vascular endothelial growth factor induces EDRF-dependent relaxation in coronary arteries. *Am. J. Physiol.* 265 H586–H592. 10.1152/ajpheart.1993.265.2.H586 8368362

[B53] Lee S. Chen T. T. Barber C. L. Jordan M. C. Murdock J. Desai S. (2007). Autocrine VEGF signaling is required for vascular homeostasis. *Cell* 130 691–703. 10.1016/j.cell.2007.06.054 17719546 PMC3010851

[B54] Li J. Cubbon R. M. Wilson L. A. Amer M. S. Mckeown L. Hou B. (2011). Orai1 and CRAC channel dependence of VEGF-activated Ca^2+^ entry and endothelial tube formation. *Circ. Res.* 108 1190–1198. 10.1161/CIRCRESAHA.111.243352 21441136 PMC3512576

[B55] Mac Gabhann F. Popel A. S. (2007). Dimerization of VEGF receptors and implications for signal transduction: a computational study. *Biophys. Chem.* 128 125–139. 10.1016/j.bpc.2007.03.010 17442480 PMC2711879

[B56] Mac Gabhann F. Qutub A. A. Annex B. H. Popel A. S. (2010). Systems biology of pro-angiogenic therapies targeting the VEGF system. *Wiley Interdiscip. Rev. Syst. Biol. Med.* 2 694–707. 10.1002/wsbm.92 20890966 PMC2990677

[B57] Marino S. Hogue I. B. Ray C. J. Kirschner D. E. (2008). A methodology for performing global uncertainty and sensitivity analysis in systems biology. *J. Theor. Biol.* 254 178–196. 10.1016/j.jtbi.2008.04.011 18572196 PMC2570191

[B58] Meyer R. D. Latz C. Rahimi N. (2003). Recruitment and activation of phospholipase Cgamma1 by vascular endothelial growth factor receptor-2 are required for tubulogenesis and differentiation of endothelial cells. *J. Biol. Chem.* 278 16347–16355. 10.1074/jbc.M300259200 12598525 PMC1459536

[B59] Milacic M. Haw R. Rothfels K. Wu G. Croft D. Hermjakob H. (2012). Annotating cancer variants and anti-cancer therapeutics in reactome. *Cancers* 4 1180–1211. 10.3390/cancers4041180 24213504 PMC3712731

[B60] Miller T. W. Soto-Pantoja D. R. Schwartz A. L. Sipes J. M. Degraff W. G. Ridnour L. A. (2015). CD47 receptor globally regulates metabolic pathways that control resistance to ionizing radiation. *J. Biol. Chem.* 290 24858–24874. 10.1074/jbc.M115.665752 26311851 PMC4598996

[B61] Neagoe P. E. Lemieux C. Sirois M. G. (2005). Vascular endothelial growth factor (VEGF)-A165-induced prostacyclin synthesis requires the activation of VEGF receptor-1 and -2 heterodimer. *J. Biol. Chem.* 280 9904–9912. 10.1074/jbc.M412017200 15637071

[B62] Olsson A. K. Dimberg A. Kreuger J. Claesson-Welsh L. (2006). VEGF receptor signalling - in control of vascular function. *Nat. Rev. Mol. Cell Biol.* 7 359–371. 10.1038/nrm1911 16633338

[B63] Papapetropoulos A. Garcia-Cardena G. Madri J. A. Sessa W. C. (1997). Nitric oxide production contributes to the angiogenic properties of vascular endothelial growth factor in human endothelial cells. *J. Clin. Invest.* 100 3131–3139. 10.1172/JCI119868 9399960 PMC508526

[B64] Rath G. M. Schneider C. Dedieu S. Rothhut B. Soula-Rothhut M. Ghoneim C. (2006). The C-terminal CD47/IAP-binding domain of thrombospondin-1 prevents camptothecin- and doxorubicin-induced apoptosis in human thyroid carcinoma cells. *Biochim. Biophys. Acta* 1763 1125–1134. 10.1016/j.bbamcr.2006.08.001 16962673

[B65] Rogers N. M. Sharifi-Sanjani M. Csanyi G. Pagano P. J. Isenberg J. S. (2014). Thrombospondin-1 and CD47 regulation of cardiac, pulmonary and vascular responses in health and disease. *Matrix Biol.* 37 92–101. 10.1016/j.matbio.2014.01.002 24418252 PMC4096433

[B66] Rohrs J. A. Sulistio C. D. Finley S. D. (2016). Predictive model of thrombospondin-1 and vascular endothelial growth factor in breast tumor tissue. *NPJ Syst. Biol. Appl.* 2:16030. 10.1038/npjsba.2016.30 28713587 PMC5507330

[B67] Ruan G. X. Kazlauskas A. (2012a). Axl is essential for VEGF-A-dependent activation of PI3K/Akt. *EMBO J.* 31 1692–1703. 10.1038/emboj.2012.21 22327215 PMC3321201

[B68] Ruan G. X. Kazlauskas A. (2012b). VEGF-A engages at least three tyrosine kinases to activate PI3K/Akt. *Cell Cycle* 11 2047–2048. 10.4161/cc.20535 22647379 PMC3368856

[B69] Sarbassov D. D. Guertin D. A. Ali S. M. Sabatini D. M. (2005). Phosphorylation and regulation of Akt/PKB by the rictor-mTOR complex. *Science* 307 1098–1101. 10.1126/science.1106148 15718470

[B70] Scheid M. P. Marignani P. A. Woodgett J. R. (2002). Multiple phosphoinositide 3-kinase-dependent steps in activation of protein kinase B. *Mol. Cell. Biol.* 22 6247–6260. 10.1128/MCB.22.17.6247-6260.2002 12167717 PMC134003

[B71] Schmeitz C. Hernandez-Vargas E. A. Fliegert R. Guse A. H. Meyer-Hermann M. (2013). A mathematical model of T lymphocyte calcium dynamics derived from single transmembrane protein properties. *Front. Immunol.* 4:277. 10.3389/fimmu.2013.00277 24065966 PMC3776162

[B72] Schneider C. A. Rasband W. S. Eliceiri K. W. (2012). NIH Image to ImageJ: 25 years of image analysis. *Nat. Methods* 9 671–675. 10.1038/nmeth.2089 22930834 PMC5554542

[B73] Shalaby F. Rossant J. Yamaguchi T. P. Gertsenstein M. Wu X. F. Breitman M. L. (1995). Failure of blood-island formation and vasculogenesis in Flk-1-deficient mice. *Nature* 376 62–66. 10.1038/376062a0 7596435

[B74] Shen W. G. Peng W. X. Dai G. Xu J. F. Zhang Y. Li C. J. (2007). Calmodulin is essential for angiogenesis in response to hypoxic stress in endothelial cells. *Cell Biol. Int.* 31 126–134. 10.1016/j.cellbi.2006.09.017 17081777

[B75] Silva H. S. Kapela A. Tsoukias N. M. (2007). A mathematical model of plasma membrane electrophysiology and calcium dynamics in vascular endothelial cells. *Am. J. Physiol. Cell Physiol.* 293 C277–C293. 10.1152/ajpcell.00542.2006 17459942

[B76] Simons M. Gordon E. Claesson-Welsh L. (2016). Mechanisms and regulation of endothelial VEGF receptor signalling. *Nat. Rev. Mol. Cell Biol.* 17 611–625. 10.1038/nrm.2016.87 27461391

[B77] Smadja D. M. D’audigier C. Bieche I. Evrard S. Mauge L. Dias J. V. (2011). Thrombospondin-1 is a plasmatic marker of peripheral arterial disease that modulates endothelial progenitor cell angiogenic properties. *Arterioscler. Thromb. Vasc. Biol.* 31 551–559. 10.1161/ATVBAHA.110.220624 21148423

[B78] Soto-Pantoja D. R. Shih H. B. Maxhimer J. B. Cook K. L. Ghosh A. Isenberg J. S. (2014). Thrombospondin-1 and CD47 signaling regulate healing of thermal injury in mice. *Matrix Biol.* 37 25–34. 10.1016/j.matbio.2014.05.003 24840925 PMC4955854

[B79] Sun Z. Li X. Massena S. Kutschera S. Padhan N. Gualandi L. (2012). VEGFR2 induces c-Src signaling and vascular permeability in vivo via the adaptor protein TSAd. *J. Exp. Med.* 209 1363–1377. 10.1084/jem.20111343 22689825 PMC3405501

[B80] Tan W. H. Popel A. S. Mac Gabhann F. (2013a). Computational model of Gab1/2-Dependent VEGFR2 Pathway to Akt Activation. *PLoS One* 8:e67438. 10.1371/journal.pone.0067438 23805312 PMC3689841

[B81] Tan W. H. Popel A. S. Mac Gabhann F. (2013b). Computational model of VEGFR2 pathway to ERK activation and modulation through receptor trafficking. *Cell. Signal.* 25 2496–2510. 10.1016/j.cellsig.2013.08.015 23993967 PMC3865527

[B82] Taraboletti G. Roberts D. Liotta L. A. Giavazzi R. (1990). Platelet thrombospondin modulates endothelial cell adhesion, motility, and growth: a potential angiogenesis regulatory factor. *J. Cell Biol.* 111 765–772. 10.1083/jcb.111.2.765 1696271 PMC2116188

[B83] Weddell J. C. Imoukhuede P. I. (2017). Integrative meta-modeling identifies endocytic vesicles, late endosome and the nucleus as the cellular compartments primarily directing RTK signaling. *Integr. Biol.* 9 464–484. 10.1039/C7IB00011A 28436498

[B84] Yao M. Rogers N. M. Csanyi G. Rodriguez A. I. Ross M. A. St Croix C. (2014). Thrombospondin-1 activation of signal-regulatory protein-alpha stimulates reactive oxygen species production and promotes renal ischemia reperfusion injury. *J. Am. Soc. Nephrol.* 25 1171–1186. 10.1681/ASN.2013040433 24511121 PMC4033366

[B85] Zhang X. Y. Birtwistle M. R. Gallo J. M. (2014). A general network pharmacodynamic model-based design pipeline for customized cancer therapy applied to the VEGFR pathway. *CPT Pharmacometrics Syst. Pharmacol.* 3 e92. 10.1038/psp.2013.65 24429593 PMC3910016

[B86] Zhao C. Isenberg J. S. Popel A. S. (2017). Transcriptional and post-transcriptional regulation of thrombospondin-1 expression: a computational model. *PLoS Comput. Biol.* 13:e1005272. 10.1371/journal.pcbi.1005272 28045898 PMC5207393

