# Supplementary material for: Computer Simulation of TSP1 Inhibition of VEGF–Akt–eNOS: An Angiogenesis Triple Threat
Source: Front Physiol. 2018 May 30;9:644. doi: 10.3389/fphys.2018.00644 (PMC5988849; doi:10.3389/fphys.2018.00644)
Supplement: Supplementary file 1 [file Table_1.DOCX]

**Table S1: List of model parameters.**

| **Parameters** | **Description** | **Value** | **Unit** |
| --- | --- | --- | --- |
| Volcyto | Cytoplasmic volume | 9.12E-13 | L |
| VolER | ER volume | 3.35E-13 | L |
| Cellarea | Cell area | 1400 | μm^2^ |
| VEGF165a_0 | VEGF concentration | 0.0012 | μM |
| VEGFR2_0 | VEGFR2 level | 4.29E+00 | #/μm^2^ |
| VEGFR1_0 | VEGFR1 level | 1.43 | #/μm^2^ |
| NRP1_0 | NRP1 level | 2.86E+01 | #/μm^2^ |
| TSP1_0 | TSP1 concentration | 0.00E+00 | μM |
| Calcium_0 | Baseline cytoplasmic calcium concentration | 0.05 | μM |
| CaM_0 | Baseline CaM concentration | 1 | μM |
| eNOS_0 | eNOS concentration | 0.1 | μM |
| kvron | The on-rate of binding VEGF to VEGFR2 | 10.3 | μM^-1^s^-1^ |
| kvroff | The off-rate for the binding of VEGF to VEGFR2 | 2.36E-01 | s^-1^ |
| kcVR | The coupling rate for the binding of VR to R through the coupling site | 0.0045 | μm^2^#^-1^s^-1^ |
| kcRR | The coupling rate between receptors | 1.11 | μm^2^#^-1^s^-1^ |
| kdRR | The decoupling rate between receptors | 0.78 | s^-1^ |
| kvr1on | The on-rate of binding of VEGF to VEGFR2 | 22 | μM^-1^s^-1^ |
| kvr1off | The off-rate of VEGF binding to VEGFR1 | 0.026 | s^-1^ |
| kdeltaRR | The coupling rate within a dimer | 2.5 | s^-1^ |
| kdeltaVR | The coupling rate between VEGF and VEGFR within a dimer | 2.05 | s^-1^ |
| kpr2 | VEGFR2 autophosphorylation rate | 30 | s^-1^ |
| kdps | Dephosphorylation rate at the surface | 640 | s^-1^ |
| kdpi | Dephosphorylation rate within signaling endosomes | 0.72 | s^-1^ |
| kdpr | Dephosphorylation rate of receptors in the recycling endosomes | 1.00E+01 | s^-1^ |
| kcd47free_on | The constitutive rate of CD47 association with VEGFR2 | 100 | μm^2^#^-1^s^-1^ |
| kr2si | VEGFR2 internalization rate in the absence of NRP1 | 0.031 | s^-1^ |
| kr2rs | VEGFR2 transport rate from recycling endosome to the surface | 17.18 | s^-1^ |
| kr2NRP1si | VEGFR2 internalization rate with NRP1 | 0.0014 | s^-1^ |
| kr2NRP1i2r | VEGFR2 transport rate from signaling endosome to recycling endosome | 48 | s^-1^ |
| kVEGFNRP1on | The on-rate for the binding of VEGF to NRP1 | 2.48 | μM^-1^s^-1^ |
| kVEGFNRP1off | The off-rate of VEGF binding to NRP1 | 0.0008 | s^-1^ |
| kNRP1VEGFR2on | The on-rate for the binding of VEGF-NRP1 to VEGFR2 | 0.15 | μm^2^#^-1^s^-1^ |
| kNRP1VEGFR2off | The off-rate for the binding of VEGF-NRP1 to VEGFR2 | 0.045 | s^-1^ |
| kNRP1VEGFR1on | The on-rate for the binding of VEGF-NRP1 to VEGFR1 | 5.07 | μm^2^#^-1^s^-1^ |
| kNRP1VEGFR1off | The off-rate for the binding of VEGF-NRP1 to VEGFR1 | 0.016 | s^-1^ |
| kVEGFR2NRP1on | The on-rate for the binding of VEGFR2 complexes to free NRP1 | 0.0022 | μm^2^#^-1^s^-1^ |
| kVEGFR2NRP1off | The off-rate for the binding of VEGFR2 complexes to free NRP1 | 0.014 | s^-1^ |
| kVEGFR1NRP1on | The on-rate for the binding of VEGFR1 complexes to free NRP1 | 0.004 | μm^2^#^-1^s^-1^ |
| kVEGFR1NRP1off | The off-rate for the binding of VEGFR1 complexes to free NRP1 | 0.1 | s^-1^ |
| PI3K_0 | The initial value of PI3K | 0.1 | μM |
| PIP2_0 | The total level of PIP2 | 10 | μM |
| kPIP2gen | The rate of PIP2 generation | 0.000048 | μMs^-1^ |
| kmPIP2PI3K | PIP2 concentration for half-maximal action of PI3K | 309.9 | μM |
| kcatPI3KPIP2 | The catalytic rate of PI3K on PIP2 | 1764.48 | s^-1^ |
| PTEN_0 | The initial concentration of the phosphatase PTEN | 0.1 | μM |
| kmPIP3PTEN | PIP3 concentration for half-maximal action of PTEN | 6.27 | μM |
| kcatPTENPIP3 | The catalytic rate of PTEN on PIP3 | 4767.44 | s^-1^ |
| konPDK1PIP3 | The on-rate for the binding of PDK1 to PIP3 | 5828.07 | μM^-1^s^-1^ |
| koffPDK1PIP3 | The off-rate for the binding of PDK1 to PIP3 | 0.64 | s^-1^ |
| konAKTPIP3 | The on-rate for the binding of Akt to PIP3 | 12.48 | μM^-1^s^-1^ |
| koffAKTPIP3 | The off-rate for the binding of Akt to PIP3 | 0.032 | s^-1^ |
| AKT_0 | The initial concentration of Akt | 0.1 | μM |
| PDK1_0 | The initial concentration of PDK1 | 0.1 | μM |
| kpAKTPDK1 | The rate of phosphorylation of Akt by activated PDK1 on T308 | 2 | μM^-1^s^-1^ |
| mTOR_0 | The initial concentration of mTOR | 0.1 | μM |
| kpmTORAKT | The rate of phosphorylation of Akt by mTOR on S473 | 2 | s^-1^ |
| kdp473AKTPPase | Dephosphorylation of S573 on Akt | 0.1 | s^-1^ |
| kdp308AKTPPase | Dephosphorylation of T308 on Akt | 0.038 | s^-1^ |
| kpPLCgamma | The rate of PLCγ activation by the active VEGFR2 population | 0.045 | μm^2^#^-1^s^-1^ |
| kdpPLCgamma | The rate of PLCγ dephosphorylation (inactivation) | 0.014 | s^-1^ |
| PLCgamma_0 | The initial concentration of PLCγ | 0.2 | μM |
| kmPIP2PLCgamma | PIP2 concentration for half-maximal action of PLCγ | 4.34 | μM |
| nDAG | The Hill coefficient for the action of PLCγ | 2.6 | - |
| kcatPLCgammaDAG | The catalytic rate of PLCγ | 0.1 | s^-1^ |
| kdeg_ip3 | Degradation rate of IP3 | 0.47 | s^-1^ |
| kdeg_DAG | Degradation rate of DAG | 0.11 | s^-1^ |
| CaER_0 | The initial calcium concentration in ER | 2.00E+03 | μM |
| Iip3Ramp | The rate of calcium release from IP3 receptors on ER | 1.31E+05 | s^-1^ |
| KmIP3R | Calcium concentration for half maximal activation of IP3R | 1.6 | μM |
| I_PMCAbar | The plasma membrane calcium pump rate | 2.02 | μMs^-1^ |
| KmPMCA | Calcium concentration for half maximal activation of PM calcium pump | 0.26 | μM |
| vSERCA | The rate of ER calcium pump | 0.39 | μMs^-1^ |
| KleakER | The calcium leak rate out of the ER into the cytoplasm | 1.00E-08 | μM^-1^s^-1^ |
| KmSERCA | Calcium concentration for half maximal activation of SERCA pump | 0.15 | μM |
| KiCa | Calcium concentration for half maximal inhibition of IP3 receptors | 1 | μM |
| KBon | The on-rate for the binding of calcium to cytoplasmic calcium buffer | 100 | μM^-1^s^-1^ |
| KBoff | The off-rate for the binding of calcium to cytoplasmic calcium buffer | 300 | s^-1^ |
| CaF_0 | Initial concentration of free cytoplasmic calcium buffer | 118 | μM |
| CaFbound_0 | Initial concentration of bound cytoplasmic calcium buffer | 2 | μM |
| koffCaNCaM1 | The off-rate for binding of calcium to the EF hand on the N lobe of CaM | 500 | s^-1^ |
| kdCaNCaM | The dissociation constant for the binding of calcium to the EF hand on the N lobe of CaM | 24 | μM |
| koffCaCCaM1 | The off-rate for the binding of calcium to the EF hand on the C lobe of CaM | 10 | s^-1^ |
| kdCaCCaM | The dissociation constant for the binding of calcium to the EF hand on the C lobe of CaM | 3.1 | μM |
| CSQN_total | Total concentration of calsequestrin (ER calcium buffer) | 15000 | μM |
| KCSQN | Dissociation constant for calcium binding to calsequestrin | 800 | μM |
| kdegi0 | Degradation rate of phosphorylated VEGFR2 with no NRP1 | 2.02E-03 | s^-1^ |
| kdegr2NRP1i0 | Degradation rate of phosphorylated VEGFR2 with NRP1 | 7.91E-03 | s^-1^ |
| kdegi0noP | Degradation rate of unphosphorylated VEGFR2 with no NRP1 | 2.85E+00 | s^-1^ |
| kdegr2NRP1i0noP | Degradation of unphosphorylated VEGFR2 with NRP1 | 2.22E-04 | s^-1^ |
| kr2ii2 | The transition rate of receptors from the first signaling endosomes to the second | 4.30E-03 | s^-1^ |
| kr2NRP1ii2 | The transition rate of receptors with NRP1 from the first signaling endosomes to the second | 4.80E+00 | s^-1^ |
| kr2i2i | The transition rate from the second signaling endosome to the first | 3.23E-02 | s^-1^ |
| kr2NRP1i2i | The transition rate of receptors with NRP1 from the second signaling endosomes to the first | 1.00E-03 | s^-1^ |
| kdegi20 | Degradation rate of phosphorylated receptors that are in the second signaling endosome | 2.43E+02 | s^-1^ |
| kdegr2NRP1i20 | Degradation rate of phosphorylated receptors with NRP1 from the second signaling endosome | 3.76E-02 | s^-1^ |
| kdegi20noP | Degradation rate of unphosphorylated receptors from the second signaling endosome | 4.16E-01 | s^-1^ |
| kdegr2NRP1i20noP | Degradation rate of unphosphorylated receptors with NRP1 from the second signaling endosome | 7.56E+02 | s^-1^ |
| ksingleR2syn | The effective synthesis rate of VEGFR2 | 1.40E-04 | s^-1^ |
| ksingleR2deg | The degradation rate of single and free VEGFR2 receptors | 5.88E-04 | s^-1^ |
| ksingleR2si | The internalization rate of single and free VEGFR2 receptors | 7.56E-04 | s^-1^ |
| ksingleR2is | The recycling rate of single and free VEGFR2 receptors | 8.66E-04 | s^-1^ |
| ICracamp | The rate of calcium entry through the CRAC channels on the plasma membrane | 5.86E+03 | μMs^-1^ |
| Kcrac | ER calcium concentration for half maximal CRAC current | 169 | μM |
| Istim0 | The initial rate of current through CRAC channels | 0.18 | μMs^-1^ |
| tau_stim | The time constant for the activation of CRAC current | 4 | s |
| ncrac | The Hill coefficient for the activation of CRAC channels by ER calcium | 4.2 | - |
| Src_0 | Initial concentration of Src | 0.1 | μM |
| Axl_0 | Initial level of Axl receptor on the membrane | 7.14 | #/μm^2^ |
| kpSrc | Rate of Src activation by phosphorylated VEGFR2 population | 0.61 | μm^2^#^-1^s^-1^ |
| kdpSrc | Rate of dephosphorylation of Src | 136.5 | s^-1^ |
| kpSrcAxl | Rate of phosphorylation of Axl by Src | 3.92 | μM^-1^s^-1^ |
| kpAxlauto | Rate of Axl autophosphorylation | 0.12 | s^-1^ |
| kdpautoAxl | Rate Axl dephosphorylation on autophosphorylated residues | 1291.1 | s^-1^ |
| kdpSrcAxl | Rate of Axl dephosphorylation on Src-phosphorylated residues | 1.79E-03 | s^-1^ |
| konPI3KAxl | Rate of PI3K activation by Axl | 55.91689 | μm^2^#^-1^s^-1^ |
| koffPI3KAxl | Rate of PI3K inactivation | 0.6 | s^-1^ |
| kDCD47TSP1 | The dissociation constant for the binding of CD47 to TSP1 | 1.00E-05 | μM |
| koffCD47TSP1 | The off-rate for the binding of CD47 to TSP1 | 0.001 | s^-1^ |
| CD47free_0 | The initial level of CD47 | 7.14E+00 | #/μm^2^ |
| kdpeNOS | Dephosphorylation rate of S1177 on eNOS | 5.17E-02 | s^-1^ |
| konCaMeNOS | The on-rate for the binding of CaM to eNOS | 9.80E+02 | μM^-1^s^-1^ |
| koffCaMeNOS | The off-rate for the binding of CaM to eNOS | 9.73E+00 | s^-1^ |
| koffeNOScav1 | The rate of eNOS unbinding from caveolin-1 | 6.875 | s^-1^ |
| koncaveNOS | The basal on-rate of eNOS binding to caveoline-1 | 81.9375 | s^-1^ |
| kcateNOSAKT | The rate of eNOS phosphorylation by Akt | 1000 | μM^-1^s^-1^ |
| kr2CD47off | The rate of TSP1-CD47 dissociation from VEGFR2 | 1.00E+00 | s^-1^ |
| f_TSP1deg | The effect of TSP1 on the degradation of receptor complexes from the signaling endosomes | 1.00E+00 |  |
| fTSP1dp | The effect of TSP1 in recruiting phosphatases to the membrane | 1.00E+00 |  |
| fTSP1i2r | The effect of TSP1 inhibiting transition from the signaling endosomes to the recycling endosomes | 1.00E+00 |  |
