# Supplementary material for: Computer Simulation of TSP1 Inhibition of VEGF–Akt–eNOS: An Angiogenesis Triple Threat
Source: Front Physiol. 2018 May 30;9:644. doi: 10.3389/fphys.2018.00644 (PMC5988849; doi:10.3389/fphys.2018.00644)
Supplement: Supplementary file 2 [file Table_2.DOCX]

**Supplementary material**

**Supplementary methods**

1. **Description of species in BioNetGen functional format**

**vegf(r,r,nrp1bd,c~s~i~i2~r)**: VEGF-A molecule

r: Binding site for the receptor

nrp1bd: NRP1 binding domain

c: The location of the VEGF-A, either surface(s), the first internalized compartment(i), the second internalized compartment (i2), the recycling compartment (r).

**vegfr2(l1,Y1175~Y~pY,CD47bd,dimer,c~s~i~i2~r)**: VEGFR2 molecule

l1: The binding site for the ligand VEGF-A

Y1175: The tyrosine phosphorylation site that can be either in phosphorylated (pY) or unphosphorylated state (Y).

CD47bd: Binding domain for CD47

dimer: Ligand-independent binding domain to VEGFR2 or VEGFR1.

c: VEGFR2 compartment either at the surface (s), the first internalized compartment (i), the second internalized compartment (i2), and the recycling compartment (r).

**vegfr1(l2,dimer,nrp1bd,c~s)**: VEGFR1 molecule

l2: the binding site for VEGF-A

nrp1bd: NRP1 binding domain

c: location of VEGFR1, assumed to be surface bound with negligible internalization

**NRP1(vegfabd,c~s~i~i2~r)**: NRP1 coreceptor molecule

vegfabd: Binding domain for VEGF-A

c: location can be either surface (s), the first internalized compartment (i), the second internalized compartment (i2), or the recycling compartment (r)

**PI(PIsite~3P~4P):**  Phosphoinositide molecule at the membrane

PIsite: phosphoinositide phosphorylation site that may be either unphosphorylated (3P) or phosphorylated (4P).

**PLCgamma(Yplc~Y~pY)**: PLCγ

Yplc: Phosphorylation site on PLCγ that can be either unphosphorylated (Y) or phosphorylated (pY)

**DAG(pkcbd)**: DAG molecule with a PKC binding domain (pkcbd)

**IP3_cyto**(): IP3 molecule

**Calcium_cyto**(): cytoplasmic calcium

**CaER(bd)**: ER calcium with a single binding domain (bd)

**CaF(cabd)**: Free calcium buffer in cytoplasm with a calcium binding domain (cabd)

**CSQNF(cabd)**: ER calcium buffer (calcequestrin) with a single binding domain for calcium (cabd)

**CaM(NCaM,NCaM,CCaM,CCaM,CaMtargetbd)**: Calmodulin with two N-terminal EF-hand domain calcium biding sites (NCaM), and two C-terminal EF-hand domain calcium binding domains (CCaM), and a binding domain for calmodulin targets (CaMtargetbd)

**Istim()**: The current through the CRAC channels

**PTEN()**: The phosphatase PTEN with a docking site for PIP3 (PIP3docking)

**TSADSrc()**: The VEGFR2 binding adaptor TSAd and the kinase SRC lumped together as TSADSrc.

**Axl(Ysrc~Y~pY,Yaxl~Y~pY)**: Axl-1 receptor with phosphorylation site for Src (Ysrc) that can be in unphosphorylated (Y) or phosphorylated (pY) state and the autophosphorylation site o Axl-1 (Yaxl) that is either in unphosphorylated (Y) or phosphorylated (pY) state.

**PI3K(state~active~inactive)**: The kinase PI3K which can either in active (state~active) or inactive (state~inactive) state

**AKT(PHakt,T308~S~pS,S473~S~pS)**: The kinase Akt with plextrin homology (PH) domain (PHakt) for binding to PIP3

**PDK1(PHpdk1)**: PDK1 with plextrin homology (PH) domain binding site for PIP3 (PHpdk1)

**mTOR()**: mTOR kinase

**CD47SIRPa(VEGFR2bd,TSP1bd,c~s~i~i2~r)**: CD47 and SIRα

**TSP1(CD47bd)**: Thrombospondin-1 with a single binding site for CD47. While TSP1 is a trimer, we simplify the model significantly by lumping together the TSP1 agent as a single agent with the effective Kd for CD47.

**eNOS(CaMBD,S1177~S~pS,cav1BD)**: eNOS with a binding domain for CaM (CaMBD), a serine phosphorylation site (S1177) which can be in either unphosphorylated (S) or phosphorylated state (pS), and a caveolin-1 binding domain (cav1BD).

**caveolin1(eNOSbd)**: caveolin-1 with a single binding domain for eNOS (eNOSbd)

1. **Coarse-grained model for the signaling pathway downstream of phosphorylated VEGFR2**

Signaling downstream of VEGFR2 phosphorylation is considered to be coarse-grained to reduce the number of species and reactions and prevent combinatorial explosion.

Receptor phosphorylation rules are as follows:

**B1.** VEGFR2 phosphorylation with the rate kpr2. Note that this occurs at other compartments as well and the condition for autophosphorylation is that the receptors are heterodimerized with VEGF:

vegf(r!1,r!2,c~s).vegfr2(l1!1,c~s).vegfr2(l1!2,Y1175~Y,c~s) -> \

vegf(r!1,r!2,c~s).vegfr2(l1!1,c~s).vegfr2(l1!2,Y1175~pY,c~s) kpr2

vegf(r!1,r!2,c~i).vegfr2(l1!1,c~i).vegfr2(l1!2,Y1175~Y,c~i) -> \

vegf(r!1,r!2,c~i).vegfr2(l1!1,c~i).vegfr2(l1!2,Y1175~pY,c~i) kpr2

vegf(r!1,r!2,c~i2).vegfr2(l1!1,c~i2).vegfr2(l1!2,Y1175~Y,c~i2) -> \

vegf(r!1,r!2,c~i2).vegfr2(l1!1,c~i2).vegfr2(l1!2,Y1175~pY,c~i2) kpr2

vegf(r!1,r!2,c~r).vegfr2(l1!1,c~r).vegfr2(l1!2,Y1175~Y,c~r) -> \

vegf(r!1,r!2,c~r).vegfr2(l1!1,c~r).vegfr2(l1!2,Y1175~pY,c~r) kpr2

**B2.** **Rules for receptor dephosphorylation**

**VEGFR2 not bound to CD47**

vegfr2(Y1175~pY,CD47bd,c~s) -> vegfr2(Y1175~Y,CD47bd,c~s) kdps

**VEGFR2 bound to CD47 but without TSP1**

vegfr2(Y1175~pY,CD47bd!3,c~s).CD47SIRPa(TSP1bd,VEGFR2bd!3,c~s) -> vegfr2(Y1175~Y,CD47bd!3,c~s).CD47SIRPa(TSP1bd,VEGFR2bd!3,c~s) kdps

**VEGFR2 bound to CD47-TSP1 with phosphatase recruitment modeled by the parameter fTSP1dp>1**

vegfr2(Y1175~pY,CD47bd!3,c~s).CD47SIRPa(TSP1bd!+,VEGFR2bd!3,c~s) -> vegfr2(Y1175~Y,CD47bd!3,c~s).CD47SIRPa(TSP1bd!+,VEGFR2bd!3,c~s) kdps*fTSP1dp

Same rule holds in other compartments:

vegfr2(Y1175~pY,CD47bd,c~i) -> vegfr2(Y1175~Y,CD47bd,c~i) kdpi

vegfr2(Y1175~pY,CD47bd!3,c~i).CD47SIRPa(TSP1bd,VEGFR2bd!3,c~i) -> vegfr2(Y1175~Y,CD47bd!3,c~i).CD47SIRPa(TSP1bd,VEGFR2bd!3,c~i) kdpi

vegfr2(Y1175~pY,CD47bd!3,c~i).CD47SIRPa(TSP1bd!+,VEGFR2bd!3,c~i) -> vegfr2(Y1175~Y,CD47bd!3,c~i).CD47SIRPa(TSP1bd!+,VEGFR2bd!3,c~i) kdpi*fTSP1dp

vegfr2(Y1175~pY,CD47bd,c~i2) -> vegfr2(Y1175~Y,CD47bd,c~i2) kdpi

vegfr2(Y1175~pY,CD47bd!3,c~i2).CD47SIRPa(TSP1bd,VEGFR2bd!3,c~i2) -> vegfr2(Y1175~Y,CD47bd!3,c~i2).CD47SIRPa(TSP1bd,VEGFR2bd!3,c~i2) kdpi

vegfr2(Y1175~pY,CD47bd!3,c~i2).CD47SIRPa(TSP1bd!+,VEGFR2bd!3,c~i2) -> vegfr2(Y1175~Y,CD47bd!3,c~i2).CD47SIRPa(TSP1bd!+,VEGFR2bd!3,c~i2) kdpi*fTSP1dp

**B3. Rule for receptor synthesis**

I() -> I() + vegfr2(l1,Y1175~Y,CD47bd,dimer,c~s) ksingleR2syn*VEGFR2total

This corresponds to the differential equation below:

$Rate=kingleR2syn\times VEGFR2_{0}$ where VEGFR2_0_ is the initial (total) VEGFR2 level. This means that ksingleR2syn is the rate of increase in fractional total VEGFR2.

**B4.** PLCγ **activation**

The rule for the activation of PLCγ by phosphorylated VEGFR2 at the surface:

PLCgamma(Yplc~Y)+vegfr2(Y1175~pY,c~s)-> PLCgamma(Yplc~pY)+vegfr2(Y1175~pY,c~s) kpPLCgamma

And other compartments:

PLCgamma(Yplc~Y) + vegfr2(Y1175~pY,c~i) -> PLCgamma(Yplc~pY) + vegfr2(Y1175~pY,c~i) kpPLCgamma

PLCgamma(Yplc~Y) + vegfr2(Y1175~pY,c~i2) -> PLCgamma(Yplc~pY) + vegfr2(Y1175~pY,c~i2) kpPLCgamma

And its dephosphorylation is given by:

PLCgamma(Yplc~pY) -> PLCgamma(Yplc~Y) kdpPLCgamma

This corresponds to the following differential equation:

$$\begin{matrix} \frac{d[pPLC\gamma]}{dt}=kpPLCgamma\times[PLC\gamma]\times([pVEGFR2]_{s}+[pVEGFR2]_{i}+[pVEGFR2]_{i2})- \\ kdpPLCgamma\times[pPLC\gamma] \end{matrix}$$

**B5. IP3 and DAG generation by activated** PLCγ

The rules in BioNetGen synthax are as follows:

PLCgamma(Yplc~pY) + PI(PIsite~3P) -> IP3_cyto() + PLCgamma(Yplc~pY) kcatPLCgammaDAG*freepip2^(nDAG-1)/(kmPIP2PLCgamma^nDAG+freepip2^nDAG)

PLCgamma(Yplc~pY) + PI(PIsite~3P) -> DAG(pkcbd) + PLCgamma(Yplc~pY) kcatPLCgammaDAG*freepip2^(nDAG-1)/(kmPIP2PLCgamma^nDAG+freepip2^nDAG)

I() -> I() + PI(PIsite~3P) kPIP2gen

IP3_cyto(ip3rbd) -> Trash() kdeg_ip3

DAG(pkcbd) -> Trash() kdeg_DAG

These correspond to the following differential equations:

$$\begin{matrix} \frac{d[IP3]}{dt}=kcaPLCgammaDAG\times[pPLC\gamma]\left(\frac{[PIP2]^{nDAG}}{kmPIP2PLCgamma^{nDAG}+[PIP2]^{nDAG}}\right)- \\ \text{kdeg\_ip3}\times\text{[IP3]} \end{matrix}$$

$$\begin{matrix} \frac{d[DAG]}{dt}=kcatPLCgammaDAG\times[pPLC\gamma]\left(\frac{[PIP2]^{nDAG}}{kmPIP2PLCgamma^{nDAG}+[PIP2]^{nDAG}}\right)- \\ \text{kdeg\_DAG}\times\text{[DAG]} \end{matrix}$$

$$\frac{d[PIP2]}{dt}=\text{kPIP2gen}$$

**B6. Calcium cycling module**

The model for calcium dynamics has been described previously ([Silva et al., 2007](#_ENREF_3);[Schmeitz et al., 2013](#_ENREF_2);[Bazzazi and Popel, 2017](#_ENREF_1)).

**B7. Activation of Src by activated VEGFR2**

The rules for the activation of TSAd-Src are as follows:

From the surface receptors:

TSADSrc(Y1~Y) + vegfr2(Y1175~pY!?,c~s) -> TSADSrc(Y1~pY) + vegfr2(Y1175~pY!?,c~s) kpSrc

From the internalized compartments:

TSADSrc(Y1~Y) + vegfr2(Y1175~pY!?,c~i) -> TSADSrc(Y1~pY) + vegfr2(Y1175~pY!?,c~i) kpSrc

TSADSrc(Y1~Y) + vegfr2(Y1175~pY!?,c~i2) -> TSADSrc(Y1~pY) + vegfr2(Y1175~pY!?,c~i2) kpSrc

TSADSrc(Y1~pY) -> TSADSrc(Y1~Y) kdpSrc

The corresponding differential equation is:

$$\begin{matrix} \frac{d[pTSAdSrc]}{dt}=kpSrc[TSAdSrc]([pVEGFR2]_{s}+[pVEGFR2]_{i}+[pVEGFR2]_{i2})-kdpSrc[pTSAdSrc] \\ \\ \end{matrix}$$

**B8. Phosphorylation of Axl-1 by Src**

TSADSrc(Y1~pY) + Axl(Ysrc~Y) -> TSADSrc(Y1~pY) + Axl(Ysrc~pY) kpSrcAxl

Rate=kpSrcAxl[TSADSrc(Y1~pY)][ Axl(Ysrc~Y)]

**B9. Axl-1 autophosphorylation**

Axl(Ysrc~pY,Yaxl~Y) -> Axl(Ysrc~pY,Yaxl~pY) kpAxlauto

Rate= kpAxlauto[Axl(Ysrc~pY,Yaxl~Y)]

**B10. Dephosphorylation of Axl-1**

Axl(Yaxl~pY) -> Axl(Yaxl~Y) kdpautoAxl

Rate= kdpautoAxl[Axl(Yaxl~pY)]

Axl(Ysrc~pY) -> Axl(Ysrc~Y) kdpSrcAxl

Rate= kdpSrcAxl[Axl(Ysrc~pY)]

**B11. Activation of PI3K by activated Axl-1**

PI3K(state~inactive) + Axl(Yaxl~pY) -> PI3K(state~active) + Axl(Yaxl~pY) konPI3KAxl

PI3K(state~active) -> PI3K(state~inactive) koffPI3KAxl

Rate for the activation of PI3K:

Rate=konPI3KAxl [PI3K(state~inactive)] [Axl(Yaxl~pY)]- koffPI3KAxl [PI3K(state~active)]

**B12. Phosphorylation of PIP2 (PI(PIsite~3P) ) to PIP3 (PI(PIsite~4P)) by activated PI3K**

PI3K(state~active) + PI(PIsite~3P) -> PI3K(state~active) + PI(PIsite~4P)

$$Rate=\frac{kcatPI3KPIP2[PIP2][PI3K(state\sim active)]}{kmPIP2PI3K+[PIP2]}$$

**B13. Dephosphorylation of PIP3 to generate PIP2**

PTEN() + PI(PIsite~4P) -> PTEN() + PI(PIsite~3P)

$$Rate=\frac{kcatPTENPIP3[PTEN][PIP3]}{kmPIP3PTEN+[PIP3]}$$

**B14. PDK1 and Akt binding to PIP3 through their PH domains**

PDK1(PHpdk1) + PI(PIsite~4P) <-> PDK1(PHpdk1!1).PI(PIsite~4P!1) konPDK1PIP3,koffPDK1PIP3

AKT(PHakt) + PI(PIsite~4P) <-> AKT(PHakt!1).PI(PIsite~4P!1) konAKTPIP3,koffAKTPIP3

**B15. Akt phosphorylation on serine 473 (S473) by mTOR with rate kpmTORAKT**

AKT(PHakt!+,S473~S) -> AKT(PHakt!+,S473~pS) kpmTORAKT

**B16. Akt phosphorylation by PDK1 on threonine 308 (T308) with rate kpAKTPDK1**

AKT(PHakt!+,S473~pS,T308~S) + PDK1(PHpdk1!+) -> \

PDK1(PHpdk1!+) + AKT(PHakt!+,S473~pS,T308~pS) kpAKTPDK1

**B17. Dephosphorylation of Akt at S473 and T308 with rate kdp473AKTPPase and kdp308AKTPPase respectively**

AKT(S473~pS) -> AKT(S473~S) kdp473AKTPPase

AKT(T308~pS) -> AKT(T308~S) kdp308AKTPPase

**B18. eNOS activation by Ca/CaM and activated Akt**

Ca/CaM binding to eNOS with rates konCaMeNOS and koffCaMeNOS respectively:

CaM(NCaM!+,NCaM!+,CCaM!+,CCaM!+,CaMtargetbd) + eNOS(CaMBD) <-> \

CaM(NCaM!+,NCaM!+,CCaM!+,CCaM!+,CaMtargetbd!1).eNOS(CaMBD!1) konCaMeNOS,koffCaMeNOS

Ca/CaM binding to eNOS leads to the dissociation of eNOS from caveoline-1 with the rate koffeNOScav1:

eNOS(CaMBD!+,cav1BD!1).caveolin1(eNOSbd!1) -> eNOS(CaMBD!+,cav1BD) + caveolin1(eNOSbd) koffeNOScav1

**B19. The basal binding of eNOS to caveolin-1**

eNOS(CaMBD,cav1BD,S1177~S) + caveolin1(eNOSbd) -> eNOS(CaMBD,cav1BD!1,S1177~S).caveolin1(eNOSbd!1) koncaveNOS

**B20. Phosphorylation of eNOS on serine 1177 (S1177) by activated AKT**

eNOS(CaMBD!+,S1177~S) + AKT(T308~pS,S473~pS) -> eNOS(CaMBD!+,S1177~pS) + AKT(T308~pS,S473~pS) kcateNOSAKT

Rate= kcateNOSAKT [eNOS(CaMBD!+,S1177~S)][ AKT(T308~pS,S473~pS)]

eNOS(S1177~pS) -> eNOS(S1177~S) kdpeNOS

Rate= kdpeNOS [eNOS(S1177~pS)]

1. **Modeling the effect of TSP1 on receptor degradation**

The parameter f_TSP1deg>1 describes the change when TSP1-CD47 is part of the receptor complex. As an example, the following rule is the degradation of the phosphorylated VEGFR2, bound to a single CD47 and TSP1:

vegf(r!1,r!2,nrp1bd,c~i).vegfr2(l1!1,Y1175~pY,CD47bd!3,c~i).CD47SIRPa(TSP1bd!+,VEGFR2bd!3,c~i).vegfr2(l1!2,CD47bd,c~i) -> Trash() kdegi0*f_TSP1deg

kdegi0 is the rate for the case without TSP1.

As another example, following is the rule describing the degradation of phosphorylated VEGFR2 bound to NRP1 and bound to a single CD47 and TSP1:

vegf(r!1,r!2,nrp1bd!9,c~i).NRP1(vegfabd!9,c~i).vegfr2(l1!1,Y1175~pY,CD47bd!3,c~i).CD47SIRPa(TSP1bd!+,VEGFR2bd!3,c~i).vegfr2(l1!2,CD47bd,c~i) -> Trash() kdegr2NRP1i0*f_TSP1deg

kdegr2NRP1i0 is the degradation rate for the case without TSP1.

1. **Modeling the effect of TSP1 on receptor transport to recycling compartment**

The parameter fTSP1i2r (between 0 and 1) models the effect of TSP1 on the translocation of the receptor complex from the internalized compartment i2 to the recycling compartment (r). For example, the rule below describes the translocation of NRP1-bound VEGFR2 with single CD47 and TSP1 bound to it:

vegf(r!1,r!2,nrp1bd!9,c~i2).NRP1(vegfabd!9,c~i2).vegfr2(l1!1,CD47bd!3,dimer,c~i2).CD47SIRPa(TSP1bd!+,VEGFR2bd!3,c~i2).vegfr2(l1!2,CD47bd,dimer,c~i2) -> \

vegf(r!1,r!2,nrp1bd!9,c~r).NRP1(vegfabd!9,c~r).vegfr2(l1!1,CD47bd!3,dimer,c~r).CD47SIRPa(TSP1bd!+,VEGFR2bd!3,c~r).vegfr2(l1!2,CD47bd,dimer,c~r) kr2NRP1i2r*fTSP1i2r

kr2NRP1i2r is the rate of transport for the case with no TSP1.

Bazzazi, H., and Popel, A.S. (2017). Computational investigation of sphingosine kinase 1 (SphK1) and calcium dependent ERK1/2 activation downstream of VEGFR2 in endothelial cells. *PLoS Comput Biol* 13**,** e1005332.

Schmeitz, C., Hernandez-Vargas, E.A., Fliegert, R., Guse, A.H., and Meyer-Hermann, M. (2013). A mathematical model of T lymphocyte calcium dynamics derived from single transmembrane protein properties. *Front Immunol* 4**,** 277.

Silva, H.S., Kapela, A., and Tsoukias, N.M. (2007). A mathematical model of plasma membrane electrophysiology and calcium dynamics in vascular endothelial cells. *Am J Physiol Cell Physiol* 293**,** C277-293.
