# Supplementary material for: Computer Simulation of TSP1 Inhibition of VEGF–Akt–eNOS: An Angiogenesis Triple Threat
Source: Front Physiol. 2018 May 30;9:644. doi: 10.3389/fphys.2018.00644 (PMC5988849; doi:10.3389/fphys.2018.00644)
Supplement: Supplementary file 3 [file Table_3.DOCX]

**Table S3: List of acronyms.**

| **Acronym** | **Description** |
| --- | --- |
| VEGF | Vascular endothelial growth factor |
| VEGFR2 | Vascular endothelial growth factor receptor type 2 |
| TSP1 | Thrombospondin-1 |
| eNOS | endothelial nitric oxide synthase |
| Ca/CaM | Calcium/Calmodulin |
| CHX | cyclohexamide |
| CRAC | Calcium release activated channels |
| Akt | Protein kinase B |
| PLCγ | Phospholipase C gamma |
| NRP1 | Neuropillin-1 |
| PTEN | Phosphatase and tensin homologue |
| PIP2 | Phosphatidylinositol 4,5-bisphosphate |
| PIP3 | Phosphatidylinositol (3,4,5)-bisphosphate |
| SERCA | Sarco/endoplasmic reticulum Ca^2+^-ATPase |
| Src | Proto-oncogene tyrosine-protein kinase Src |
| Axl-1 | Axin-like protein 1 |
| PI3K | Phosphoinositide 3-kinase |
| CD47 | Cluster of differentiation 47 |
| CD36 | Cluster of differentiation 36 |
